# Cloning, Expression and Characterization of UDP-Glucose Dehydrogenases

**DOI:** 10.3390/life11111201

**Published:** 2021-11-07

**Authors:** Márcia R. Couto, Joana L. Rodrigues, Lígia R. Rodrigues

**Affiliations:** Centre of Biological Engineering, University of Minho, 4710-057 Braga, Portugal; marcia.couto@ceb.uminho.pt (M.R.C.); lrmr@deb.uminho.pt (L.R.R.)

**Keywords:** UDP-glucose dehydrogenase, UDP-glucuronic acid, glycosaminoglycans biosynthesis, heterologous production, *Escherichia coli*, *Saccharomyces cerevisiae*

## Abstract

Uridine diphosphate-glucose dehydrogenase (UGD) is an enzyme that produces uridine diphosphate-glucuronic acid (UDP-GlcA), which is an intermediate in glycosaminoglycans (GAGs) production pathways. GAGs are generally extracted from animal tissues. Efforts to produce GAGs in a safer way have been conducted by constructing artificial biosynthetic pathways in heterologous microbial hosts. This work characterizes novel enzymes with potential for UDP-GlcA biotechnological production. The UGD enzymes from *Zymomonas mobilis* (*Zm*UGD) and from *Lactobacillus johnsonii* (*Lbj*UGD) were expressed in *Escherichia coli*. These two enzymes and an additional eukaryotic one from *Capra hircus* (*Ch*UGD) were also expressed in *Saccharomyces cerevisiae* strains. The three enzymes herein studied represent different UGD phylogenetic groups. The UGD activity was evaluated through UDP-GlcA quantification in vivo and after in vitro reactions. Engineered *E. coli* strains expressing *Zm*UGD and *Lbj*UGD were able to produce in vivo 28.4 µM and 14.9 µM UDP-GlcA, respectively. Using *S. cerevisiae* as the expression host, the highest in vivo UDP-GlcA production was obtained for the strain CEN.PK2-1C expressing *Zm*UGD (17.9 µM) or *Ch*UGD (14.6 µM). Regarding the in vitro assays, under the optimal conditions, *E. coli* cell extract containing *Lbj*UGD was able to produce about 1800 µM, while *Zm*UGD produced 407 µM UDP-GlcA, after 1 h of reaction. Using engineered yeasts, the in vitro production of UDP-GlcA reached a maximum of 533 µM using *S. cerevisiae* CEN.PK2-1C_pSP-GM_*Lbj*UGD cell extract. The UGD enzymes were active in both prokaryotic and eukaryotic hosts, therefore the genes and expression chassis herein used can be valuable alternatives for further industrial applications.

## 1. Introduction

Uridine diphosphate-glucose dehydrogenase (UDP-glucose dehydrogenase, UGD, EC 1.1.1.22) is an oxidoreductase that catalyzes the production of UDP-glucuronic acid (UDP-GlcA) from UDP-glucose (UDP-Glc). UDP-GlcA is a compound with diverse applications whose major biotechnological use is as a precursor for glycosaminoglycans (GAGs) production [1].

Glycosaminoglycans (GAGs) are a class of natural long linear polysaccharides that include hyaluronic acid, heparan sulfate, and chondroitin sulfate which have medical, veterinary, pharmaceutical, and cosmetic applications. Nevertheless, except for hyaluronic acid that is produced by microbial fermentation, GAGs are mainly extracted from animal sources using laborious methods and hazardous reagents, generating products with heterogeneous compositions, raising concerns of adulteration and risks of prion contamination, which ultimately limits their large-scale production [2]. Additionally, religious, and ethical issues alongside with vegetarianism trends are key factors driving the search for non-animal sources of GAGs. An eco-friendly and controllable alternative consists of the metabolic engineering of microbial cell factories to produce GAGs from simple carbon sources [3,4,5].

**Figure 1:**
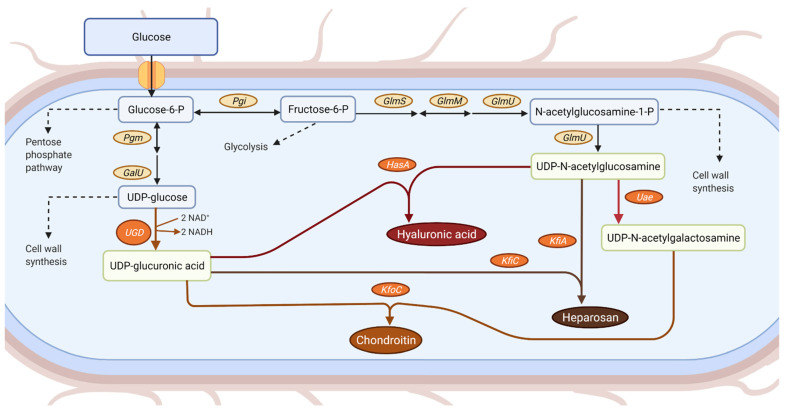
Production of uridine diphosphate (UDP)-glucuronic acid in *Escherichia coli* and its possible use in the biosynthesis of microbial chondroitin, hyaluronic acid, or heparosan. Depending on the microbial host, the heterologous expression of the enzymes shown in orange might be required for glycosaminoglycans production. Enzyme abbreviations: GalU, uridine-triphosphate:glucose-1-phosphate uridylyltransferase; GlmM, phosphoglucosamine mutase; GlmS, glucosamine-6-phosphate synthase; GlmU, glucosamine-1-phosphate N-acetyltransferase/N-acetylglucosamine-1-phosphate uridyltransferase; HasA, hyaluronan synthase; KfiA, β-1,3-glucuronyltransferase; KfiC, α-1,4-*N*-acetylglucosaminyltransferase; KfoC, chondroitin synthase; Pgi, glucose-6-phosphate isomerase; Pgm, phosphoglucomutase; Uae, UDP-*N*-acetylglucosamine 4-epimerase; UGD, UDP-glucose 6-dehydrogenase.

Several pathogenic microorganisms have been reported as GAG-like polysaccharides producers through well-known pathways (Figure 1). For example, group A and C streptococci and *Pasteurella multocida* type A produce hyaluronic acid [6,7]; *Escherichia coli* K5, *P. multocida* type D and *Avibacterium paragallinarum* genotype II can synthesize a capsular polysaccharide, heparosan, structurally analogous to unsulfated heparin [8,9,10]; *E. coli* O5:K4:H4 synthesizes a capsular polysaccharide composed by unsulfated fructosylated chondroitin [8]; and *P. multocida* type F and *A. paragallinarium* genotype I produce unsulfated chondroitin [9,10]. However, the biotechnological production of GAGs at an industrial-scale, and especially of sulfated GAGs, is limited, due to low yields and safety issues related to the cultivation of pathogenic microorganisms [11]. Therefore, in the past few years research efforts have been focused on the improvement of the production process, as well as on the design of better heterologous hosts to produce GAGs. Recent advances in the microbial production of GAGs using non-pathogenic hosts (mostly prokaryotic organisms) have been reported [12,13,14,15,16,17,18,19].

The heterologous pathway design should take into account every enzymatic step to maximize GAGs yields. The step catalyzed by UGD was previously considered to be the limiting factor of GAG biosynthesis in homologous and heterologous organisms [13,16,20]. Since the first discovered UGD [21], genes encoding UGD have been identified in almost all organisms, from which alternative genes can be extracted for further evaluation as biocatalytic producers of UDP-GlcA. However, although UGD genes are widespread across nature, few UGD have been biochemically characterized or evaluated in heterologous hosts. The overexpression of UGD enzyme in a heterologous host is needed even in hosts containing the gene as it is often over-regulated limiting its expression [22]. Additionally, no eukaryotic heterologous host has yet been evaluated for the production of some GAGs. Eukaryotic hosts can be a favorable alternative for eukaryotic gene expression offering an interesting platform for complex integrated production and sulfation systems.

In this study, the expression of three novel genes encoding UGD was evaluated to expand the knowledge of potential candidates for the construction of GAGs biosynthetic pathways. Two prokaryotic UGDs from *Zymomonas mobilis* (*Zm*UGD) and *Lactobacillus johnsonii* (*Lbj*UGD) were expressed in *E. coli* BL21 (DE3). In addition, a eukaryotic system was also used for heterologous expression of these UGD enzymes to assess if this chassis could be a proper platform for GAGs production. *Saccharomyces cerevisiae* CEN.PK2-1C and *S. cerevisiae* BY4741 were used to express *Zm*UGD and *Lbj*UGD and an additional UGD from *Capra hircus* (*Ch*UGD) that has never been evaluated. These enzymes are representatives of each of the three evolutionary groups identified by an evolutionary analysis, namely from group I and II of prokaryotic UGD and from the eukaryotic group. The expressed UGD enzymes were characterized for the first time in a prokaryotic and eukaryotic host through in vivo and in vitro reactions by quantification of UDP-GlcA production. These enzymes were found to have a high catalytic efficiency and to be suitable for use in the construction of biosynthetic pathways for GAGs production. In addition, *S. cerevisiae* was demonstrated to be a suitable alternative platform for UDP-GlcA production.

## 2. Materials and Methods

### 2.1. Multiple Sequence Alignment and Evolutionary Relationships

The evolutionary relationship of the three UGD enzymes explored in this work, *Ch*UGD, *Zm*UGD, and *Lbj*UGD (GenBank sequences XP_013820045.1 WP_012817132.1, ABM21411.1, respectively), was accessed. Clustal Omega (1.2.4) tool of European Molecular Biology Laboratory—European Bioinformatics Institute (EMBL-EBI, https://www.ebi.ac.uk/Tools/msa/clustalo/, accessed on 3 November 2021) was used to perform a multiple amino acid sequences alignment of the three enzymes and their domains were predicted using the HMMER web service (EMBL-EBI, https://www.ebi.ac.uk/Tools/hmmer/search/phmmer, accessed on 3 November 2021). Using *Zm*UGD amino acid sequence as query, a sequence similarity search was performed using BLASTp tool at National Center for Biotechnology Information (NCBI, https://blast.ncbi.nlm.nih.gov/, accessed on 3 November 2021), against the reviewed proteins in the SwissProt/UniProt database. The identified homologous protein sequences and 5 additional relevant protein sequences from known GAG producers (a total of 63 representative enzymes) were analyzed through multiple sequence alignment (ClustalW algorithm) and evolutionary analysis in MEGA-X [23]. The phylogenetic tree was constructed in MEGA-X using the Neighbor-Joining method [24]. The Poisson correction method was used to compute evolutionary distances [25] which are in the units of the number of amino acid substitutions per site. All ambiguous positions were removed for each sequence pair (pairwise deletion option).

### 2.2. Strains and Plasmids

The strains and plasmids used in this study are listed in Table 1. *Z. mobilis* ATCC 29191 and *L. johnsonii* ATCC 11506 were obtained from the American Type Culture Collection (ATCC, Manassas, VA, USA) and used as genomic DNA (gDNA) source for amplification of UGD genes. *E. coli* NZY5α (NZYTech, Lisbon, Portugal) was used for cloning procedures and vector propagation. *E. coli* BL21 (DE3) (NZYTech) was used as expression host of *Zm*UGD or *Lbj*UGD genes using pRSFDuet-1 (Novagen, Madison, WI, USA) as the expression vector. *S. cerevisiae* CEN.PK2-1C and *S. cerevisiae* BY4741 strains were obtained from Euroscarf (Oberursel, Germany) and used for UGD expression. In this case, two different shuttle vectors were also tested, namely pSP-GM1 (PGK1 promoter, Addgene, Watertown, MA, USA) and p426GPD (ATCC).

### 2.3. Gene Sources and Cloning Strategy

Table S1 compiles the primers used for gene cloning. UGD genes from *Z. mobilis* and *L. johnsonii* (GenBank accession numbers CP003704.1:c550193-548880 and EF138834.1:15898-17145, respectively) were amplified from gDNA. Phusion High Fidelity DNA Polymerase (Thermo Fisher Scientific, Wilmington, United States) was used for all gene amplifications unless otherwise stated. Amplification of the *Zm*UGD gene from the *Z. mobilis* genome was achieved directly through colony Polymerase Chain Reaction (PCR) using the primers Zm_Fw and Zm_Rv. The gDNA from the Gram-positive *L. johnsonii* was extracted from a 2 mL culture using Wizard^®^ Genomic DNA Purification Kit (Promega, Madison, WI, USA) according to the manufacturer instructions with slight modifications as follows: the pellet was treated only with 10 mg/mL of lysozyme prior nuclei lysis; in the end of the procedure, the DNA was rehydrated with 50 µL of DNA Rehydration Buffer and stored at 4 °C. This DNA was used as template for *Lbj*UGD gene amplification with primers Lbj_Fw and Lbj_Rv. Since the amplification yield was low, the cleaned PCR product was further A-tailored to be cloned in Promega’s pGEM^®^-T Easy Vector and the final construction was used as amplification template. A-tailored product was achieved by adding 5 units of Dream *Taq* DNA Polymerase (Thermo Scientific) to the PCR product and 0.2 mM dNTPs in a final volume of 10 µL and incubating the mixture at 70 °C for 30 min. Then, 1 µL of this reaction was added to a ligation reaction with Promega’s pGEM^®^-T Easy Vector according to the kit instructions originating pGEM_*Lbj*UGD. UGD gene from *C. hircus* (GenBank accession number XM_018049379.1) was codon-optimized for *S. cerevisiae* and synthesized by NZYTech (Table S2). Afterwards, it was amplified from pUC57_*Ch*UGD using the Ch_Fw and Ch_Rv primers. After amplification, *Zm*UGD, *Lbj*UGD, *and Ch*UGD genes were cloned in p426GPD and pSP-GM1. To clone *Zm*UGD and *Lbj*UGD genes in pRSFDuet-1, the genes were amplified from p426GPD_*Zm*UGD and pGEM_*Lbj*UGD using the Zm_prsf_FW/Zm_prsf_Rv and Lbj_prsf_Fw/Lbj_prsf_Rv primer pairs, respectively.

All genes were cloned in frame with a N-terminal 6× Histidine tag originally present in pRSFDuet-1 or in the primers used to clone the genes in pSP-GM1 and p426GPD.

Plasmid DNA was extracted using NucleoSpin^®^ Plasmid Miniprep Kit (Macherey-Nagel, Düren, Germany). Amplified DNA fragments were purified from agarose using NucleoSpin^®^ Gel and PCR Clean-up Kit (Macherey-Nagel). Plasmid DNA and PCR products were quantified using a NanoDrop instrument (ND-1000, Thermo Fisher Scientific) and were digested with the proper restriction endonucleases (Thermo Fisher Scientific) for 1 h at 37 °C and purified using NucleoSpin^®^ Gel and PCR Clean-up Kit. Ligations were performed for 1 h at room temperature using a T4 DNA ligase (Thermo Fisher Scientific). The constructions were transformed by heat shock into *E. coli* NZY5α competent cells (NZYTech). Super optimal broth with catabolite repression (SOC; NZYTech) was used for transformants recovery. In the case of pGEM_*Lbj*UGD construction, positive transformants were selected using the blue-white screening method. White colonies were picked from lysogeny broth (LB) (10 g/L tryptone, 5 g/L yeast extract, 10 g/L NaCl; NZYTech) agar plates (20 g/L agar, JMGS, Odivelas, Portugal) containing ampicillin (0.1 mg/mL, NZYTech), 5-bromo-4-chloro-3-indolyl-β-D-galactopyranoside (X-Gal, 0.1 mg/mL; NZYTech) and isopropyl β-D-1-thiogalactopyranoside (IPTG, 0.5 mM; NZYTech). All plasmids herein constructed were verified by colony PCR using Dream *Taq* polymerase and digestion and their sequences were further confirmed by sequencing (GATC Biotech, Konstanz, Germany). After sequence confirmation, *E. coli* BL21 (DE3) competent cells (NZYTech) were transformed with the constructed plasmids pRSFDuet_*Zm*UGD, pRSFDuet_*Lbj*UGD and empty pRSFDuet-1 (used as negative control). Transformations of *S. cerevisiae* were performed by lithium acetate/single-stranded carrier DNA/polyethylene glycol method [29]. Lithium acetate, salmon sperm DNA and polyethylene glycol (PEG-3350) were obtained from Sigma-Aldrich. Selection of transformants was performed in selective minimal medium Yeast Nitrogen Base (YNB) with ammonium sulfate without amino acids (Sigma-Aldrich, St. Louis, MI, USA) with required amino acids in the absence of uracil.

### 2.4. Culture Conditions

*Z. mobilis* was grown at 30 °C in solid medium with glucose 20 g/L (Acros Organics, Morris Plains, NJ, USA), yeast extract 5 g/L (Panreac AppliChem, Darmstadt, Germany) and agar 15 g/L. *L. johnsonii* was grown in de Man, Rogosa and Sharpe (MRS) medium (Himedia, Mumbai, India) at 37 °C and 200 rpm.

*E. coli* cultures were incubated at 37 °C and 200 rpm in LB, or on LB agar plates. Ampicillin at a final concentration of 100 µg/mL (NZYTech) or kanamycin at 50 µg/mL (Fisher Scientific, Springfield, NJ, USA) were supplemented when necessary. For UGD enzyme expression and consequent UDP-GlcA production, engineered *E. coli* BL21 (DE3) were grown by inoculating 1% (*v*/*v*) of an overnight pre-culture in 50 mL LB medium supplemented with kanamycin in 250 mL Erlenmeyer flasks (initial optical density at 600 nm (OD_600nm_) = 0.1), which were then shaken at 37 °C and 200 rpm. *E. coli* transformed with the empty vector, pRSFDuet-1, was also grown and used as negative control. When an OD_600nm_ of 0.6 was reached, IPTG was added at a final concentration of 1 mM. Cells were then grown at 30 °C and 200 rpm for additional 3 h. The absorbance was measured in a 96-well plate spectrophotometric reader Synergy HT (BioTek, Winooski, VT, USA). Dry weight of cells was determined through a calibration curve with OD_600 nm_ and expressed as g/L.

Wild-type *S. cerevisiae* growth was performed at 30 °C, 200 rpm, in Yeast Extract Peptone Dextrose (YPD, 20 g/L bacteriological peptone (HiMedia), 10 g/L yeast extract, 20 g/L glucose) or in plates with same composition supplemented with agar. After transformation, cells were grown in YNB plates supplemented with 20 g/L glucose and the required amino acids depending on the strain (tryptophan, histidine, methionine (Panreac AppliChem) and leucine (Fisher Scientific) and, in the case of wild-type strains, the pyrimidine uracil (Sigma)), at final concentrations of 100 mg/L. A single colony was picked from the plate and grown 24 h in 8 mL YNB for pre-culture. Afterwards, 50 mL of the selective minimal medium YNB supplemented with the required amino acids in 250 mL flasks were inoculated to an initial OD_600 nm_ of 0.1. Cells were grown at 30 °C and 200 rpm for 24–30 h.

### 2.5. Preparation of E. coli and S. cerevisiae Protein Extracts

In the end of the fermentation, the final culture of *E. coli* cells (~50 mL) was centrifuged (5000× *g* 15 min) and the pellet resuspended in 10 mL of Tris–HCl buffer (10 mM, pH 7.8). Cells were then lysed by sonication with a microtip probe linked to Vibra-cell processor (Sonics, Newtown, CT, USA). Keeping the solution on ice during the procedure, short pulses of 3 s ON and 9 s OFF at 30% amplitude were performed until 5 min of active sonication was reached. The resulting lysate was centrifuged (12,000× *g* 15 min). The soluble fraction was collected and filtered with 0.2 µm NY filter and the insoluble phase was saved separately after resuspending the pellet in 1 mL of 10 mM Tris–HCl buffer. The filtered soluble samples were analyzed to quantify in vivo UDP-GlcA production through ultra-high-performance liquid chromatography (UHPLC) and were further used to perform in vitro reactions.

Cells from *S. cerevisiae* grown cultures (~50 mL) were harvested by centrifugation (5000× *g* 15 min). For each 0.1 g of wet cells, 0.2 g of glass beads (425–600 µm, Sigma-Aldrich) were added to the cells pellet, as well as 2 mL of Tris–HCl (10 mM, pH 7.8) and 1× protease inhibitor cocktail (NZYTech). FastPrep-24 (MP Biomedicals, Salon, USA) was then used during 5 cycles of 1 min at 6–6.5 m/s interspersed with 1 min cooling on ice. Lysed samples were centrifuged (16,000× *g* 15 min) and the supernatant was filtered with 0.2 µm filter. Soluble and insoluble fractions were kept separately. The soluble fraction samples were analyzed using UHPLC to quantify in vivo UDP-GlcA production and were used for in vitro reactions.

Protein concentration in the soluble and insoluble fractions was measured using Pierce™ Coomassie (Bradford) Protein Assay Kit according to the manufacturers’ instructions.

The UGD expression of *E. coli* and *S. cerevisiae* was evaluated by sodium dodecyl sulfate polyacrylamide gel electrophoresis (SDS–PAGE) [30] (4% stacking gel and 12% running gel). Samples were mixed with 2× Laemmli Sample Buffer (65.8 mM Tris–HCl pH 6.8, 2.1% SDS, 26.3% glycerol, 0.01% bromophenol blue, from Fisher Scientific, JMGS, and Sigma-Aldrich, respectively) with β-mercaptoethanol (AppliChem) and were denatured at 95 °C for 5 min. The protein marker used was Color Protein Standard—Broad Range (NEB, #77125) or NZYColour Protein Marker II (NZYTech). After electrophoresis, the gel was stained using Coomassie Blue R-250 (AppliChem) for 15 min and de-stained using distilled water.

### 2.6. Purification of UGD

Purification of engineered 6× His-tagged UGD from *E. coli* cell extracts was performed through affinity chromatography using nickel NTA agarose resin (ABT, Doral, FL, USA) in 2 mL Pierce™ centrifuge columns (Thermo Scientific) according to ABT’s instructions. The first elution was performed with 250 mM imidazole (Merck, Kenilworth, NJ, USA) followed by a second and third elution with 500 mM imidazole. Purified protein was quantified again with the Bradford Protein Assay Kit and analyzed by SDS–PAGE.

### 2.7. UGD In Vitro Assays

UGD enzymatic activity was evaluated through in vitro reactions using the different cell extracts as enzyme source. The in vitro reaction was set by mixing 150 µL of reaction mixture with 50 µL of cell extracts from *E. coli* or *S. cerevisiae* up to a final concentration of 0.25 g/L protein. The reaction mixture was composed by 2.5 mM UDP-Glc (Carbosynth, Compton, Berkshire, UK), 0.5 mM nicotinamide adenine dinucleotide (NAD^+^) (Panreac AppliChem), 1× protease inhibitor and 50 mM Tris–HCl pH 8.6, final concentrations in the 200 µL total volume. Immediately after adding the reaction mixture to the cell extract, the microplate was incubated at 30 °C for 60 min during which NADH (reduced form of NAD^+^) formation was monitored in a microplate reader. A calibration curve was performed measuring the absorbance at 340 nm of different NADH (Panreac AppliChem) concentrations (0–750 µM) in a UV-transparent 96-well polystyrene microplate (Sigma-Aldrich). To evaluate the optimal pH of recombinant UGDs, cell extracts from UGD-producing recombinants were incubated in reaction solutions with different pHs (from 3.5 to 9.1) for 1 h at 30 °C. To evaluate the optimal temperature, cell extracts were reacted at different temperatures (from 15 to 40 °C) with a pre-heated reaction mixture of pH 8.0 during 1 h. To determine the kinetic parameters of the UGD enzymes, the cell extract at a final protein concentration of 0.5 g/L was incubated with a pre-heated reaction mixture (0.5 mM NAD^+^, 1× protease inhibitor and 50 mM Tris–HCl pH 8.0, at 30 °C) with variable concentrations of UDP-Glc (1–5 mM). Samples were taken at several timepoints during the incubation (0–15 min). The kinetic parameters K_m_ and V_max_ were calculated by fitting the curve through nonlinear regression to the Michaelis-Menten model using GraphPad Prism 8.

### 2.8. Analysis and Quantification of UDP-Sugars

UHPLC analysis was performed to quantify UDP-sugars in bacteria and yeasts’ cell extracts (in vivo production) or reacted supernatants from in vitro assays. Analysis was performed using the Shimadzu Nexera-X2 system (Shimadzu Corporation, Kyoto, Japan) (CBM-20A system controller, LC-30AD pump unit, DGU-20A 5R degasser unit, SPD-M20A detector unit, SIL-30AC autosampler unit, CTO-20AC column oven) and the column Luna^®^ Omega 3 µm Polar C18 (150 mm × 4.6 mm, Phenomenex, Torrance, CA, USA). The column was equilibrated at 25 °C in 20 mM tetraethylammonium acetate (pH 6.0) during 8 min followed by a 22 min gradient from 0 to 5% of acetonitrile. The injection volume was 5 µL and the working flow rate was 0.5 mL/min. UDP-sugars were detected at UV_260nm_ absorbance. Retention times of UDP-Glc and UDP-GlcA were 23.8 min and 27.9 min, respectively.

Calibration curves based on UDP-Glc and UDP-GlcA standards (Carbosynth), in concentrations ranging from 50 to 2000 µM, were performed to quantify UDP-sugars in the samples.

### 2.9. Statistical Analysis

GraphPad Prism was used for all statistical analysis and the statistical significance of the differences between results was based on 2-way ANOVA with multiple comparisons (Tukey’s or Durnett’s multiple comparisons test).

## 3. Results

### 3.1. Sequences and Phylogenetic Analysis

Despite the wide range of biological sources known to express UGD, there are few studies exploring their cloning or biochemical, structural, and functional characterization. UniProt entries for this enzyme include 45,705 unreviewed and only 44 reviewed sequences (data retrieved on 4 October 2021). The enzymes used in this study, *Zm*UGD, *Lbj*UGD and *Ch*UGD, are part of the unreviewed group. None of them has been expressed in heterologous hosts or studied for biotechnological applications. Expressing these UGD representatives in both prokaryotic and eukaryotic hosts can give new insights e.g., into the gene selection for biotechnological production of GAGs. Moreover, the selection of *Ch*UGD as a eukaryotic representative enzyme was based on the fact that the highest reported specific activity of UGD was obtained using an isolated UGD from caprine liver [31].

UGD belongs to the UDP-glucose/guanosine diphosphate (GDP)-mannose 6-dehydrogenase (UGD/GMD) superfamily. These enzymes have a wide range of functions, often involved in the biosynthesis of polysaccharides and are frequently part of operons dedicated to that purpose. The sequences of the three enzymes herein studied were aligned (Figure S1) and their identity was calculated. Percent identity matrix showed that *Lbj*UGD shares 25% identity with *Ch*UGD and 30% identity with *Zm*UGD, while *Ch*UGD and *Zm*UGD exhibit 37% identify. Afterwards, *Zm*UGD sequence was screened against the proteins in the SwissProt database using BLASTp which demonstrated that this enzyme shares high levels of identity with other enzymes in the UGD/GMD superfamily. The sequences of the proteins identified as similar to *Zm*UGD, putative UGDs used in this study and UGDs from well-known GAG producers were analyzed through multiple sequence alignment. The resulting 63 representative amino acid sequences included 29 bacterial UGD, 1 archaeal UGD, 19 eukaryotic UGD, 5 GMD, 7 UAMAD, 1 UAGAD, and 1 NAGAAD. The multiple sequence analysis allowed to identify highly conserved amino acids in UGD/GMD superfamily and specifically within UGDs (highlighted residues in Figure S1).

Although the amino acid sequences can be very variable within species, all the secondary structural features were found to be conserved across the UGD/GMD superfamily sequences, with the largest sequence diversity occurring close to the C-terminus. The three UGD enzymes cloned in this work presented, as predicted, the described conserved domains, namely the NAD(^+^)-binding domain, the central domain, and UDP-sugar binding domain for the N-terminal, central, and C-terminal regions, respectively (Figure S1). Regarding the specific active site of UGD, the multiple sequences alignment confirmed that all sequences of the enzymes evaluated have the conserved residues required for UGD activity.

Afterwards, the sequences of each of the three studied enzymes were compared with NCBI sequences using BLASTp. These BLASTp results are described in Table S3. The BLASTp analysis showed that *Zm*UGD is significantly similar to the UGD from *Sinorhizobium meliloti*, followed by the UGDs from *Bacillus subtilis* subsp. *subtilis* (YwqF and TuaD), and UGD from *Pseudomonas aeruginosa*. Interestingly, these UGDs are from known polysaccharide producers. Although *Z. mobilis* itself is a recognized levan producer [32], *S. melitoti* is known to produce succinoglycan [33], *B. subtilis* is also able to synthesize levan, teichuronic acid and other exopolymeric substances [34,35], and *P. aeruginosa* produces alginate [36]. Remarkably, *Zm*UGD exhibited a higher identity with archaeal UGD from *Haloferax volcanii*, eukaryotic UGDs (such as from *Drosophila melanogaster*, *Homo sapiens*, *Pongo abelii*, *Bos taurus* and *Mus musculus*), and even with GMDs, than with other prokaryotic UGDs such as the heparosan producer *E. coli* K5. The probiotic *L. johnsonii* produces the polysaccharide inulin [37,38], and its UGD showed the highest identity with UGD from *E. coli* O111:H^−^. This strain, as well as the ones with the following greater identity are also known producers of capsular polysaccharides, including GAGs, namely *E. coli* O8:K40, *E. coli* CFT073/O6:K2:H1, *E. coli* O157:H7, *E. coli* K-12, *Salmonella enterica* and *Streptococcus pyogenes* [39,40,41,42]. Expectably, *Ch*UGD was highly identical to UGDs from other mammals (identity from 99.8 to 96% with UGDs from *B. taurus*, *H. sapiens*, *P. abelli*, *M. muscullus* and *R. novergicus*). The following enzymes with similar sequences are UGDs from plants (59.8 to 61.8% identity with *Glycine max*, *Arabidopsis thaliana* and *Oryza sativa*) followed by the group of enzymes most similar to *Zm*UGD.

**Figure 2:**
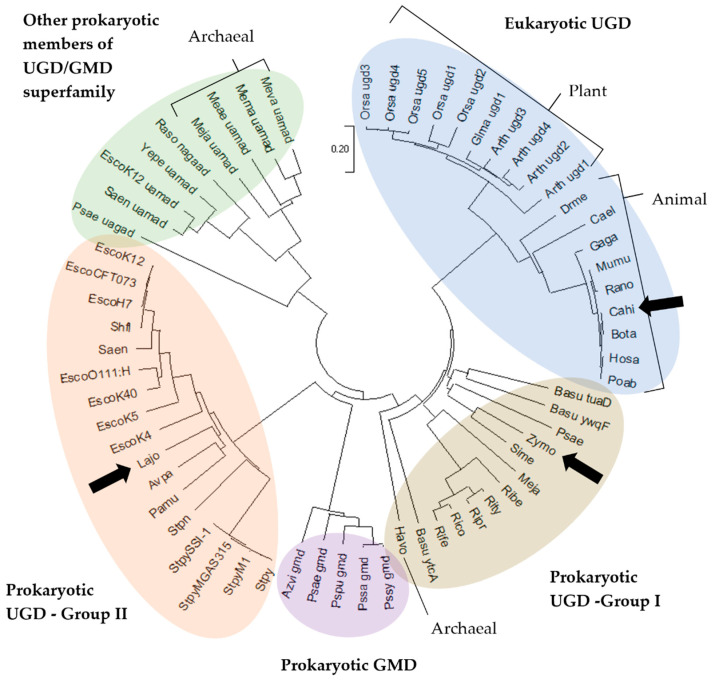
Evolutionary relationship between 63 uridine diphosphate (UDP)-glucose 6-dehydrogenase (UGD)-glucose/guanosine diphosphate (GDP)-mannose 6-dehydrogenase (GMD) superfamily members. The resulting optimal tree has the sum of branch length = 12.04551231. The tree is drawn to scale, with branch lengths corresponding to the evolutionary distances used to infer the phylogenetic tree. The dataset comprised a total of 991 sites. Enzymes are labelled by the two first letters of the genus followed by two letters of species name. The three UGD expressed in this study are indicated with a black arrow (Lajo, *Lactobacillus johnsonii*; Cahi, *Capra hircus*; and Zymo, *Zymomonas mobilis*). Abbreviation of the protein is also shown after species name when not UGD, or when multiple UGD isoforms exist within same species. Protein abbreviations: gmd, GDP-mannose 6-dehydrogenase; uamad, UDP-*N*-acetyl-D-mannosamine dehydrogenase; uagad, UDP-*N*-acetylglucosamine 6-dehydrogenase; and nagaad, nucleotide diphosphate (NDP)-*N*-acetyl-*D*-galactosaminuronic acid dehydrogenase.

Finally, a phylogenetic tree was built to show the evolutionary distances between the 63 aligned amino acid sequences (Figure 2). Five main groups of enzymes emerged, namely eukaryotic UGD, prokaryotic UGD—Group I, prokaryotic UGD—Group II, prokaryotic GMD and other prokaryotic members of UGD/GMD superfamily. The enzymes used in this study, indicated in Figure 2 with black arrows, are representative of three different phylogenetic groups of UGDs. *Lbj*UGD belongs to the group II prokaryotic UGDs, *Zm*UGD belongs to group I prokaryotic UGDs, while *Ch*UGD belongs to eukaryotic UGDs group. Granja et al. [43] previously reported the divergence between the two groups of prokaryotic UGDs. Enzymes belonging to the so-called group I are more related to eukaryotic UGDs than to the group II prokaryotic UGDs. This analysis gives insights on which enzymes exhibit functional similarities, demonstrating that *Lbj*UGD is the most similar to microbial GAG producers, *Zm*UGD is included in groups of other types of polysaccharides producing organisms, while *Ch*UGD is included in the animal GAGs producers.

### 3.2. E. coli as UGD Expression Host

**Figure 3:**
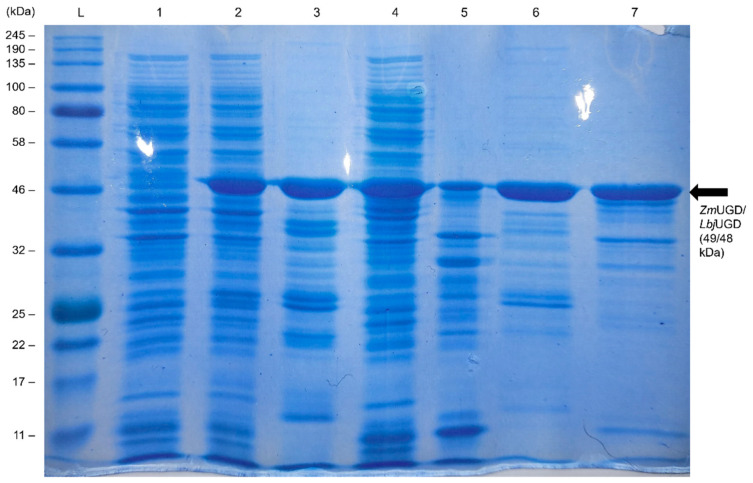
SDS–PAGE gel showing 6-His-tagged uridine diphosphate-glucose dehydrogenases (UGDs) expression in *Escherichia coli* BL21. Lane L on the left corresponds to the ladder Color Prestained Protein Standard, Broad Range (11–245 kDa). Samples loaded were: (**1**) soluble cell extract of *E. coli* pRSFDuet-1 (15 µg), (**2**) soluble cell extract from *E. coli* pRSFDuet_*Zm*UGD (15 µg), (**3**) 1st eluted fraction from *Zm*UGD purification (5 µg), (**4**) soluble cell extract from *E. coli* pRSFDuet_*Lbj*UGD (15 µg), (**5**) 1st eluted fraction from *Lbj*UGD purification (5 µg), (**6,7**) 2nd eluted fractions from *Zm*UGD and *Lbj*UGD purification, respectively (5 µg).

Since there are several reports on eukaryotic UGDs expressed in *E. coli* that were not active [44,45,46,47], only the two prokaryotic genes were here expressed in *E. coli* BL21 (DE3). This host was evaluated for the expression of *Zm*UGD and *Lbj*UGD by SDS–PAGE (Figure 3). Based on their amino acid sequence, the expected sizes for the recombinant 6× His *Zm*UGD and 6× His *Lbj*UGD were calculated to be 49.2 kDa and 48.0 kDa, respectively.

As it can be observed, *Zm*UGD and *Lbj*UGD were efficiently overexpressed in *E. coli* and exhibited the correct size. After UGD purification, as expected, the fraction from the 2nd elution showed a cleaner band.

**Figure 4:**
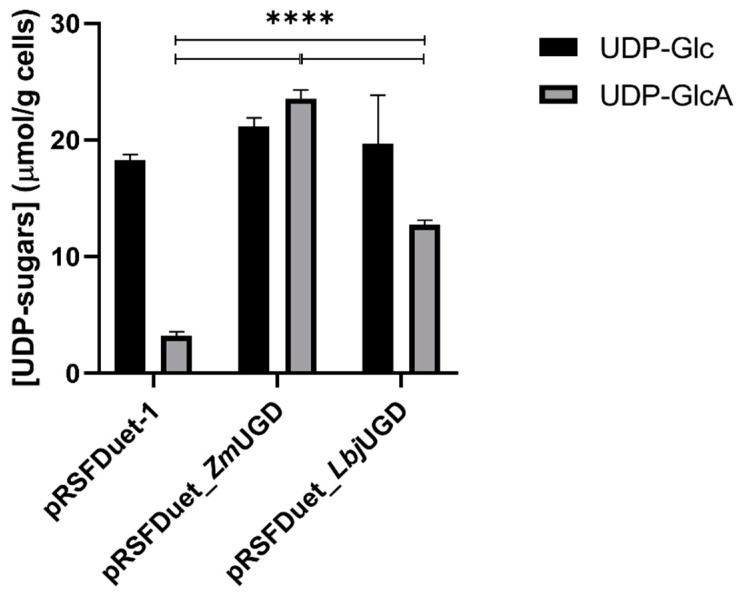
In vivo uridine diphosphate-glucose (UDP-Glc) and UDP-glucuronic acid (UDP-GlcA) production by *Escherichia coli* BL21 harboring pRSFDuet-1, pRSFDuet_*Zm*UGD or pRSFDuet_*Lbj*UGD, the last two expressing an extra copy of UDP-Glc dehydrogenase (UGD) from *Zymomonas mobilis* (*Zm*UGD) and *Lactobacillus johnsonii* (*Lbj*UGD), respectively. Results are normalized by the cultures’ dry weight of cells at the end of the fermentation. The fermentations were performed in triplicate. Four asterisks (****) identify statistically significant differences with adjusted *p* values < 0.0001.

The UDP-GlcA production in vivo by the engineered *E. coli* was quantified by UHPLC. Figure 4 shows the UDP-Glc and UDP-GlcA quantification in bacterial cell extracts, normalized by the dry cell weight obtained at the end of the fermentation.

UDP-Glc is naturally produced from glucose in *E. coli* BL21. The UDP-Glc in this strain can be used for the biosynthesis of trehalose, lipid A-core, cellulose, or for galactose degradation via the Leloir pathway. Although there is still no wet lab confirmation, the gene prediction computational methods by protein homology suggest that this strain contains a UGD gene (NCBI sequences NC_012892 region: 1994244-1995410; NC_012971 region: 1994250-1995416), which justifies the presence of small amounts of UDP-GlcA in the negative controls. Additionally, it is known that *E. coli* BL21 has a polymyxin resistance pathway [48] that requires the use of UDP-GlcA. Additionally, there is evidence that UDP-Glc regulates the activity of enzymes in *E. coli*. UDP-Glc enhances adenosine diphosphate-sugar pyrophosphatase activity, which can lead to reduced glycogen production by the cell [49].

When recombinant UGDs were expressed in *E. coli* BL21 cells, UDP-GlcA formation became evidently higher in their cell extracts compared to the one carrying the empty vector. The highest UDP-GlcA in vivo production at the end of fermentation was achieved by *E. coli* expressing *Zm*UGD, 28.4 µM (or 23.6 µmol·g^−1^ cell dry weight, CDW), while the *E. coli* expressing *Lbj*UGD produced 14.9 µM (or 12.7 µmol·g^−1^ CDW). Interestingly, the UDP-GlcA production did not result in a reduction of the UDP-Glc pool in the engineered *E. coli*. In the work of Cimini et al. [50] the UDP-sugars levels were accessed in wild-type and in an *E. coli* K4 engineered strain. UDP-Glc varied approximately from 0.1 to 0.4 µmol·g^−1^ CDW while UDP-GlcA varied from 0.1 to 1.2 µmol·g^−1^ CDW, depending on the strain and on the time of fermentation. Restaino et al. [8] reported that UDP-sugars concentrations during microbial fermentations of the pathogenic *E. coli* K4 or *E. coli* K5 in different media varied between 0.25 and 11.0 μM·g^−1^ CDW (from 0.2 to 12.0 μM), depending on the strain, type of precursor, growth phase, and culture conditions. Specifically, using *E. coli* K4 grown in a glycerol-based medium, UDP-Glc concentrations varied from 8.1 to less than 0.5 μM·g^−1^ CDW and UDP-GlcA from 0.25 to approximately 0.48 μM·g^−1^ CDW. When using *E. coli* K5 in the same medium, UDP-Glc and UDP-GlcA concentrations varied from around 11 to 1 μM·g^−1^ CDW and from almost undetectable to approximately 1.5 μM·g^−1^ CDW, respectively. The authors showed that the UDP-precursors availability considerably influenced the total chondroitin or heparosan produced. Fermentation using recombinant strains of *S. zooepidemicus* [51] resulted in the same range of UDP-sugar precursors in the exponential phase. UDP-Glc varied from approximately 1.1 to 3.9 µmol·g^−1^ CDW and UDP-GlcA varied from almost 0 to 17.2 µmol·g^−1^ CDW, according to the strain. It is important to notice that in the referred studies there is not much accumulation of UDP-GlcA as in the current work because it is being redirected for GAGs production. Nevertheless, the results from the present work show that the *E. coli* BL21 was able to produce UDP-GlcA in the same range as the reported for GAGs-producing pathogenic strains.

**Figure 5:**
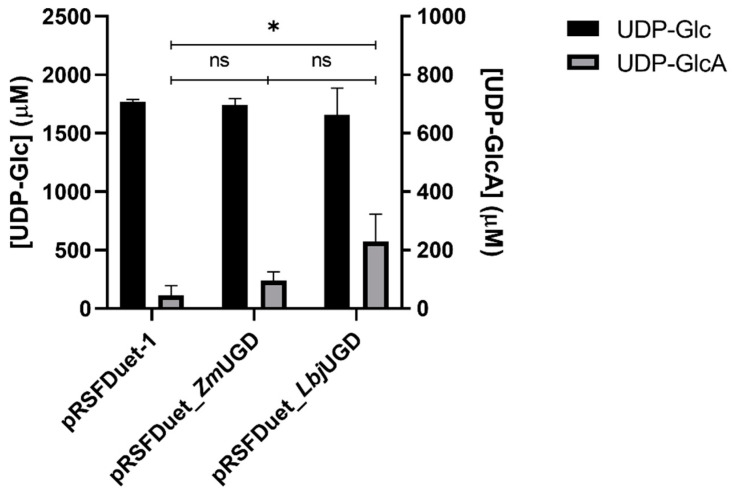
In vitro uridine diphosphate-glucose (UDP-Glc) consumption and UDP-glucuronic acid (UDP-GlcA) production by cell extracts expressed as concentration by UDP-Glc dehydrogenase (UGD) from *Zymomonas mobilis* (*Zm*UGD) and *Lactobacillus johnsonii* (*Lbj*UGD) expressed in *Escherichia coli* BL21. Values from triplicate reactions. One asterisk (*) identifies adjusted *p* values < 0.05.

After evaluating UDP-GlcA productions in vivo, cell extracts were used to perform in vitro reactions that were analyzed by UHPLC (Figure 5). Cell extracts were used because after His-Tag purification, recombinant UGDs showed no activity in vitro (data not shown).

Differently from the in vivo results, in the in vitro assay using cell extracts, the enzyme *Lbj*UGD resulted in the highest UDP-GlcA production, despite not being statistically different than *Zm*UGD. The variations from in vitro and in vivo reactions may be explained by the different pH and temperature conditions at which the assays are conducted. After 1 h of reaction, the cell extract containing *Lbj*UGD led to a 229 µM production in vitro, while the one containing *Zm*UGD led to a 95 µM production under the same conditions. Differences on substrate and product concentrations between reactions of *Lbj*UGD recombinant enzyme and negative control were statistically significant at a 95% confidence level. The initial UDP-Glc concentration was 2.5 mM and from Figure 5 it is possible to conclude that more UDP-Glc was consumed than the one required to produce the detected amounts of UDP-GlcA. This was observed also in the negative control which again shows the ability of the enzymes from *E. coli* BL21 to use UDP-Glc as substrate for competitive reactions, such as for the biosynthesis of trehalose or lipid A-core, and the ability of endogenous UGD to convert UDP-Glc to UDP-GlcA that can be afterwards metabolized in the polymyxin pathway or for colanic acid biosynthesis.

The NADH production was also evaluated through the monitoring of the absorbance at 340 nm (Figure S2) during the in vitro reactions. Absorbance increases with the NADH production. For each mol of UDP-Glc that is converted into UDP-GlcA by UGD, 2 mol of NADH are produced. However, NADH is not a suitable indicator of UGD enzymatic activity in crude cell extracts since it can also be produced by other enzymes. Nevertheless, the highest NADH productions were obtained in the reactions using cell extracts from engineered *E. coli* expressing *Lbj*UGD, as observed by the quantification of UDP-GlcA by UHPLC.

*S. cerevisiae* was further used as expression host for UGD in an attempt to achieve higher enzymatic activities and consequently higher UDP-GlcA titers.

### 3.3. Three Novel UGDs Expressed in Yeasts

In this work, the budding yeast was evaluated as an expression system for UGD enzymes since the use of eukaryotic hosts could improve the solubility and activity of the expressed enzymes. In particular, eukaryotic enzymes that are not easily expressed in *E. coli* can become viable alternatives for the construction of biosynthetic pathways.

Besides the same two prokaryotic genes previously expressed in *E. coli*, one eukaryotic UGD, codon-optimized for *S. cerevisiae*, was evaluated for UDP-GlcA production. The expression of the UGDs, fused to a His-Tag, was evaluated through SDS–PAGE (Figure S3). The transformed *S. cerevisiae* strains did not show an increased UGD expression compared to the other proteins in their cell extract and to the controls. The expected sizes of *Ch*UGD, *Zm*UGD, and *Lbj*UGD enzymes were 55.9 kDa, 48.2 kDa and 47.4 kDa, respectively. The purification of the his-tagged proteins was performed but the proteins could not be observed in the gels and the amounts produced were not enough to further use in in vitro assays. Although the constitutive promoters used (phosphoglycerate kinase, PGK1, or glyceraldehyde-3-phosphate dehydrogenase, GPD, promoters) are widely applied for high constitutively expression of recombinant proteins in yeasts [52,53,54,55], the expression is not comparable to the prokaryotic *E. coli* system where the exceptionally strong T7 promoter and a high copy number plasmid were used.

**Figure 6:**
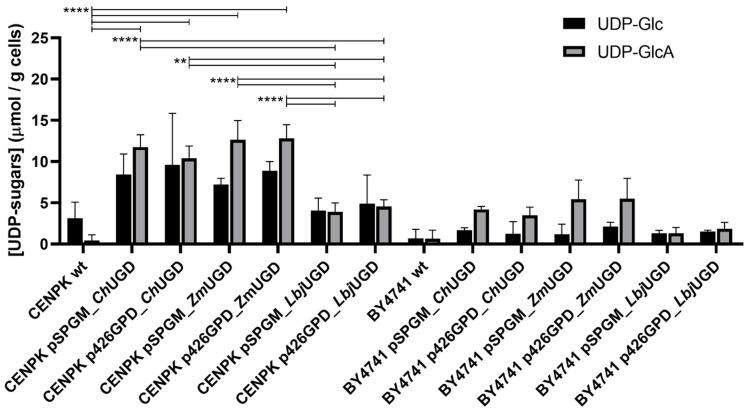
In vivo uridine diphosphate (UDP)-sugars production by *Saccharomyces cerevisiae* CEN.PK2-1C and BY4741 wild types (wt.) and engineered strains expressing UDP-glucose dehydrogenase (UGD) from *Capra hircus* (*Ch*UGD), *Zymomonas mobilis* (*Zm*UGD) and *Lactobacillus johnsonii* (*Lbj*UGD). Standard deviations are relative to at least three different transformed colonies tested. Differences in the UDP-glucuronic acid (UDP-GlcA) concentrations between the mutants and the correspondent wild-type are indicated, with statistical significance represented with two asterisks (**) for adjusted *p* values < 0.01 and with four asterisks (****) for *p* values < 0.0001. Statistically non-significative differences are not shown for simplification.

The cell extracts were analyzed through UHPLC and UDP-GlcA, although in very small amounts, was detected for all engineered *S. cerevisiae* strains. Contrarily to *E. coli*, *S. cerevisiae* does not harbor the UGD gene in its genome. Therefore, UDP-GlcA was not detected in cell extracts of wild-type yeasts. The in vivo production of UDP-GlcA by *S. cerevisiae* expressing recombinant ChUGD, *Lbj*UGD and *Zm*UGD is shown in Figure 6.

It has been reported that in *S. cerevisiae*, UDP-Glc is a naturally abundant substrate in the cytoplasm, required for the synthesis of β-glucan polysaccharides [56], but also for trehalose and glycogen biosynthesis. The UDP-Glc concentration herein obtained ranged from 1.3 to 9.9 µM (0.7 to 8.4 µmol·g^−1^ CDW), depending on the strain and plasmid used. In general, within each strain, differences between UDP-Glc concentration of all the different combinations were not statistically significant. In terms of UDP-GlcA production, all mutants showed increased UDP-GlcA production, due to UGD overexpression, as compared to the negative controls. However, differences were not statistically significative in *S. cerevisiae* BY4741 engineered strains compared to the wild-type strain. It is important to mention that as episomal plasmids were used as expression vectors, different yeast transformed colonies can have huge differences in terms of plasmid copy number and, consequently, in product concentration. Hence, future integration in the genome, as single or multiple gene copies, could result in more reproducible effects [57].

*S. cerevisiae* CEN.PK2-1C harboring pSP-GM_*Ch*UGD, p426GPD_*Ch*UGD, pSP-GM_*Zm*UGD and p426GPD_*Zm*UGD showed the highest statistically significant production compared to the wild-type, producing on average 14.6, 10.5, 17.9 and 15.9 µM, respectively (corresponding to 13.1, 9.7, 13.5 and 13.6 µmol·g^−1^ CDW). As it can be observed, the engineered strains expressing the eukaryotic enzyme *Ch*UGD and the ones expressing the prokaryotic enzyme *Zm*UGD exhibited greater UDP-GlcA amounts, while the systems expressing *Lbj*UGD generated lower UDP-GlcA levels in vivo. This observation suggests that UGD enzymes from the eukaryotic and group I prokaryotic phylogenetic groups (Figure 2) have improved in vivo activity compared to group II prokaryotic UGDs. The work of Oka & Jigami [56] was the only one that so far reported the use of *S. cerevisiae*, specifically the strain W303a, to express UGD. The expression of one of the UGD isoforms from *A. thaliana*, allowed to obtain an in vivo UDP-GlcA production of 5.71 ± 1.06 µmol·g^−1^ CDW, and the substrate UDP-Glc was reduced 54% (from 2.21 µmol·g^−1^ CDW to 1.20 µmol·g^−1^ CDW) compared to the wild-type. This decrease in UDP-Glc pool was not observed in the current work. Nevertheless, the values of UDP-GlcA herein achieved are 2 to 3 times higher (depending on the strain) than the ones obtained by those authors. In a different work, Zhou et al. [47] expressed a fungal UGD using a different yeast as host, namely *Pichia pastoris*. However, no values of UDP-GlcA production are reported in this study.

The slightly reduced in vivo UDP-GlcA production obtained with *S. cerevisiae* compared to *E. coli* might be due to the absence of a UGD homolog gene in *S. cerevisiae* as it occurs in bacteria.

**Figure 7:**
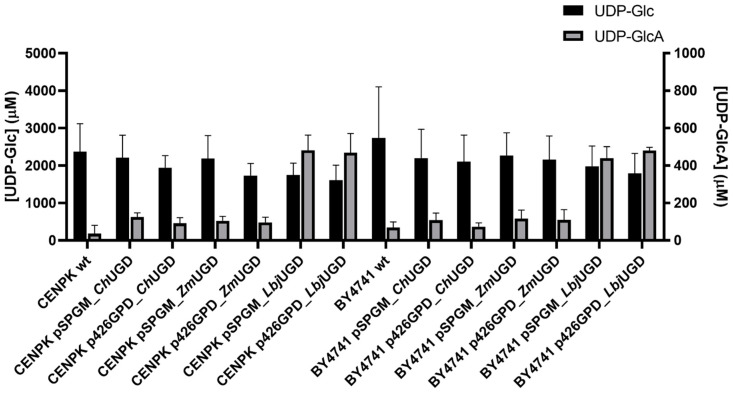
In vitro conversion of uridine diphosphate-glucose (UDP-Glc) into UDP-glucuronic acid (UDP-GlcA) by *Saccharomyces cerevisiae* CEN.PK2-1C and BY4741 cell extracts through the action of expressed UDP-Glc dehydrogenases (UGDs) from *Capra hircus* (*Ch*UGD), *Zymomonas mobilis* (*Zm*UGD) and *Lactobacillus johnsonii* (*Lbj*UGD). Standard deviations are relative to at least three independent assays.

Afterwards, the in vitro UDP-GlcA production was evaluated. Figure 7 shows the UHPLC results obtained from the in vitro reactions using the yeast cell extracts.

Although the differences in UDP-GlcA concentrations were not statistically significant, *Lbj*UGD in yeast cell extracts exhibited the highest UDP-GlcA concentrations in vitro, resembling what was observed in the in vitro reactions using *E. coli* cell extracts. The higher final UDP-GlcA concentrations obtained were on average: 482 µM using *S. cerevisiae* CEN.PK2-1C pSP-GM_*Lbj*UGD; 480 µM using BY4741 p426GPD_*Lbj*UGD; 469 µM using CEN.PK2-1C p426GPD_*Lbj*UGD; and 439 µM with BY4741 pSP-GM_*Lbj*UGD. The high standard deviation values are related to the use of cell extracts from different transformed colonies for the in vitro reactions. As it can be observed, the UDP-GlcA production in vitro was not significantly affected using different vectors or promoters for UGD expression. Using plasmids pSP-GM1 and p426GPD, containing PGK1 and GPD promoters, respectively, for UGD expression did not seem to result in statistically significative differences in UDP-GlcA production in vitro.

The NADH production was also evaluated through the monitoring of the absorbance at 340 nm (Table S4). The highest NADH productions were obtained in the reactions using cell extracts from engineered yeasts expressing *Lbj*UGD, which is in agreement with the obtained results of UDP-GlcA production.

The obtained results for the in vitro reactions using yeast cell extracts resembled the ones obtained with *E. coli* cell extracts. The enzyme that generally led to the highest UDP-GlcA concentration in vitro in engineered yeast, *Lbj*UGD, was not the one that allowed the highest production of UDP-GlcA in vivo. Again, in vivo and in vitro conditions, such as the pH, were different which may justify the different relative activities found to the enzymes.

Comparing the prokaryotic and eukaryotic hosts for UGD expression, while the highest in vivo UDP-GlcA production was achieved with engineered *E. coli*, the enzymes expressed in *S. cerevisiae* exhibited higher UDP-GlcA production in vitro. This suggests that the conditions used for in vivo UDP-GlcA production in yeasts are probably not appropriate for optimal UGD activity. For example, the pH of yeast cell extracts at the end of fermentation was slightly lower (around 6.0) than the pH of cells extracts obtained after culturing *E. coli* (around 7.0), and UGDs usually have an optimal activity at basic pH as further discussed below. Therefore, if an in vivo strategy is envisaged, *S. cerevisiae* fermentation needs to be further optimized.

It was demonstrated that *Zm*UGD was the enzyme that more actively converted UDP-Glc to UDP-GlcA in vivo in both prokaryotic and eukaryotic hosts. In the yeast, *Ch*UGD allowed to produce the same UDP-GlcA levels as *Zm*UGD. However, cell extracts containing *Lbj*UGD produced the highest UDP-GlcA, in both prokaryotic and eukaryotic hosts. Contrarily to what might be expected, the activities in vivo and in vitro of *Zm*UGD and *Lbj*UGD were very distinct, regardless of the host, despite being both from prokaryotic sources; being *Zm*UGD and *Ch*UGD more similar in terms of enzymatic activities as shown by UDP-GlcA production. This can be related to the fact that *Zm*UGD sequence is more similar to *Ch*UGD than to *Lbj*UGD as observed from the phylogenetic analysis (Figure 2).

### 3.4. Biochemical Characterization of Cloned UGDs

**Figure 8:**
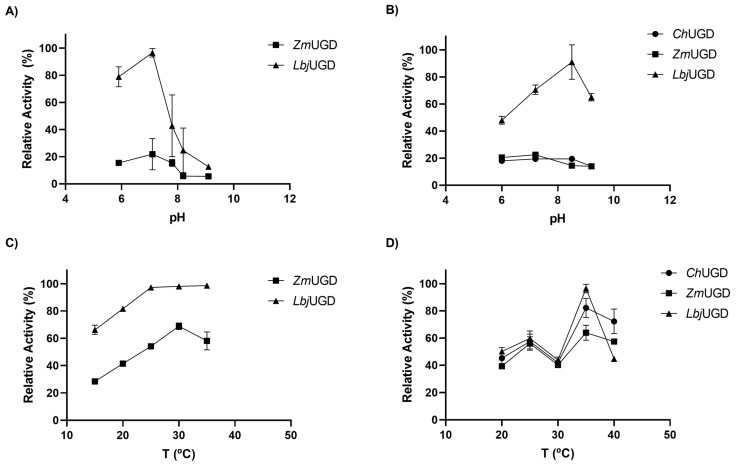
Characterization of recombinant uridine diphosphate-glucose dehydrogenases (UGDs) at different pH and temperature. In vitro reactions were performed using cell extracts containing recombinant UGD expressed in *Escherichia coli* BL21 (DE3) under T7 promoter control, on different pH at 30 °C and on different temperatures at pH 8.0, respectively (**A**,**C**) and cell extracts containing recombinant UGDs expressed in *Saccharomyces cerevisiae* CEN.PK2-1C under PGK1 promoter, on different pH at 30 °C and on different temperatures at pH 8.0, respectively (**B**,**D**). The 100% activity corresponds to the highest activity of (**A**) 31.1 × 10^−3^ U/mL, (**B**) 9.8 × 10^−3^ U/mL, (**C**) 8.4 × 10^−3^ U/mL and (**D**) 3.9 × 10^−3^ U/mL, in all cases achieved when the UGD from *Lactobacillus johnsonni* (*Lbj*UGD) was expressed. One unit of UGD activity was defined as the amount of enzyme that produces 1 μmol of uridine diphosphate-glucuronic acid per minute.

The cell extracts from *E. coli* and *S. cerevisiae* were used to determine the optimal pH and temperature for UGD recombinant enzymes under in vitro conditions. Figure 8 shows the in vitro relative enzymatic activity between different cell extracts, when varying the pH (Figure 8A,B) and the temperature (Figure 8C,D), according to the final UDP-GlcA concentration determined by UHPLC. The *S. cerevisiae* strain used for UGD expression was CEN.PK2-1C as it was the strain that previously led to higher productions in vivo and the results in vitro of both strains were similar. The genes were expressed under control of the PGK1 promoter as no significant differences among the two promoters tested were previously observed.

Cell extracts from engineered *E. coli* were used and the two expressed UGD enzymes, *Zm*UGD and *Lbj*UGD, showed slightly different properties (Figure 8A,C). Again, *Lbj*UGD allowed to produce more UDP-GlcA in vitro than *Zm*UGD. Regarding the different pH, higher UDP-GlcA content was obtained at the pH 7.1. The optimal temperature for *E. coli* expressed *Zm*UGD was 30 °C while the *Lbj*UGD conserved its maximal activity between 25 °C and 35 °C. From the UGDs expressed in *S. cerevisiae*, *Lbj*UGD generally exhibited the highest activity in vitro under different pH and temperatures (Figure 8B,D). Its activity was maximal at the temperature of 35 °C and a pH of 8.5. The other UGDs showed lower activities under all pH tested and exhibited the maximal activity at 35 °C.

Nevertheless, these results are in accordance with the reported studies on UGD activities from other organisms. For example, the pH optimum for *Cryptococcus laurentii* UGD activity was in the range of 7.3–7.8 [58]; the optimum pH and temperature of soybean UGD were reported to be 8.7 and 30 °C [59]; optimal pH for UGD activity of sugarcane was 8.4 [60]; UGD from *Sphingomonas elodea* exhibited optimum pH and temperature of 8.7 and 37 °C [43]; the UGD from the fungus *Phoma herbarum* showed the highest activity at a pH of 8.6 and at 35 °C [47]; *P. multocida’s* UGD activity was optimal at a pH of 10 and 37 °C [7]; and UGDs from *Granulibacter bethesdensis* and *Akkermansia muciniphila* showed optimal activity at 37 °C and pH 9.0 [61,62].

Expectably, these results show a general increase in UDP-GlcA concentration compared to the in vitro assays previously performed under non-optimal conditions. In these experiments, UDP-GlcA concentrations using cell extracts from *E. coli* expressing *Lbj*UGD and *Zm*UGD reached on average 1800 µM and 407 µM, respectively (pH 7.1 and 30 °C, Figure 8A). When using cell extracts from engineered *S. cerevisiae* strains, the maximum UDP-GlcA concentration achieved with *Ch*UGD and *Zm*UGD was 116 µM and 134 µM (pH 7.2 and 30 °C, Figure 8B), while *Lbj*UGD allowed to obtain 533 µM (pH 8.5 and 30 °C, Figure 8B).

This biochemical characterization of UGDs could explain the differences previously obtained in in vivo and in vitro UDP-GlcA production using the different expression systems. It was observed that UGD enzymes produced more UDP-GlcA in vivo using *E. coli* than *S. cerevisiae*. That result could be justified by the acidic environment of yeast fermentation being far from the optimal pH of UGDs. Additionally, it is demonstrated that the *Lbj*UGD activity is highly affected by the pH, so in the buffered in vitro reactions it has shown a much better performance than in vivo where pH is changing with the fermentation time. Additionally, in *E. coli* the expression temperature was 30 °C which was optimal for the enzymes expressed in that system (Figure 8C). However, the temperature of 30 °C, used for *S. cerevisiae* fermentation, was not the optimal for UGDs expressed in that chassis (Figure 8D).

Moreover, in the case of the in vitro reactions performed, presented in Section 3.2 and Section 3.3, it is important to notice that they were performed at 30 °C and at a pH of 8.0 which was here demonstrated to be close to, but not the optimal conditions for UGDs expressed in *E. coli* and in *S. cerevisiae*, thus suggesting that the UDP-GlcA production could be further improved. The kinetic parameters (K_m_ and V_max_) (Table 2). were also determined under these conditions.

As it can be seen through the V_max_/K_m_ ratio, the enzyme that showed the highest potential was *Lbj*UGD both in *E. coli* and *S. cerevisiae* systems, which agrees with the previous observed results from in vitro reactions that showed that *Lbj*UGD was able to produce more UDP-GlcA in the assay conditions. Since the kinetic parameters of characterized UGD in the literature are generally calculated with purified enzyme, it was expected that the parameters herein estimated would be significantly different from previous reports. Since the protein concentration in the cell extracts accounts for much more proteins than UGD, the reaction maximum velocities of the expressed UGDs were lower and required higher concentrations of UDP-Glc to obtain half of the maximum velocity (higher K_m_). This effect is obviously more pronounced in the enzymes expressed in yeasts since, as mentioned before, the expression of enzymes was not as evident as the ones expressed in *E. coli*, where the overexpression was easily observed in the SDS-PAGE gels. Examples of reported UGD kinetic parameters are: K_m_ of 230 µM and a V_max_ of 8.0 × 10^3^ μmol·min^−1^·g_protein_^−1^ for UGD from *Burkholderia cepacia* [63]; K_m_ and V_max_ of 110 µM and 9.8 × 10^9^ μmol·min^−1^·g_protein_^−1^, respectively, for UGD from *P. multocida* [7]; an apparent K_m_ of 137.2 μM [64] for a recombinant UGD from *Bacillus cereus* expressed in *E. coli*; and a K_m_ of 34 μM and a V_max_ of 313 μmol·min^−1^·g_protein_^−1^ for the human UGD recombinantly expressed in *E. coli* [65].

## 4. Conclusions

Although UGD is considered a bottleneck step for GAGs biotechnological production, few alternative UGDs have been characterized and evaluated for such purpose. In addition, the use of eukaryotic hosts to express enzymes from the GAG production pathway has been absent in the literature although they are candidate systems that could possibly address the low solubility and activity of recombinant enzymes.

Herein, three UGD enzymes (*Zm*UGD, *Lbj*UGD and *Ch*UGD) were expressed in *S. cerevisiae* and two prokaryotic enzymes (*Zm*UGD and *Lbj*UGD) were expressed in *E. coli*.

The sequences of the UGDs in this study were compared with other reviewed enzymes using bioinformatic tools and a phylogenetic analysis was performed, being the three genes cloned in this study representative of three UGD phylogenetic groups. *Zm*UGD was found to be functionally closer to *Ch*UGD than to the other prokaryotic *Lbj*UGD.

The activity of the UGDs was evaluated through quantification of UDP-sugars production in vivo in both *E. coli* and *S. cerevisiae* cell extracts, with and without expressing the recombinant UGD. *E. coli* was found to be a useful system for protein expression since the recombinant proteins *Zm*UGD and *Lbj*UGD were clearly overexpressed in a soluble form and a significant increase in the UDP-GlcA pool in cell extracts of engineered *E. coli* was observed as compared to the negative control. However, in vitro, the UDP-GlcA concentration did not increase very significantly. Moreover, the same two prokaryotic enzymes and one eukaryotic UGD, codon-optimized for *S. cerevisiae* (*Ch*UGD), were evaluated for UDP-GlcA production, using two different yeast strains and expression vectors. When using *S. cerevisiae* as the heterologous host, the expression of recombinant enzymes was not demonstrated in SDS–PAGE. However, the quantification by UHPLC demonstrated that all engineered yeasts produced more UDP-GlcA in vivo than the control strains. The strains with highest in vivo UDP-GlcA production were *S. cerevisiae* CEN.PK2-1C expressing *Zm*UGD or expressing *Ch*UGD. Although the highest in vivo UDP-GlcA production was achieved with engineered *E. coli*, the enzymes expressed by *S. cerevisiae* exhibited higher performance in vitro. The cell extracts were further used to determine the optimum pH and temperature for the UGD recombinant enzymes under in vitro conditions. Generally, the optimum conditions that maximize the UGD enzymes activity were a pH between 7 and 9 and a temperature between 30 and 35 °C. Remarkably, the enzyme that generally showed highest activity in vitro in both *E. coli* and yeast mutants, *Lbj*UGD, was not the one that allowed the highest production of UDP-GlcA in vivo, thus suggesting that the in vitro and in vivo conditions were sufficiently different leading to different relative activities. Depending on the strategy intended for GAGs production, i.e., in vivo or enzymatic production, different enzymes should be used, according to their kinetics, towards higher product yields.

This work evaluates alternative genes for biotechnological applications, including GAGs microbial production, recombinant protein production for enzymatic production of GAGs or to produce expensive UDP-precursors for further chemical and/or enzymatic production of GAGs. These novel UGD genes showed to efficiently convert UDP-Glc into UDP-GlcA (up to 74% conversion using *Lbj*UGD in the optimal conditions) and should be explored for the construction of a complete heterologous pathway for in vivo GAG production. Additionally, metabolic engineering of host microorganisms for improving UDP-Glc availability should be considered, either by overexpressing the pathway genes and regulators and/or by performing the knockout of competitive reactions (essentially cell wall recycling pathways) to improve intermediate pools and subsequent availability.

## Figures and Tables

**Table 1:** Strains and plasmids used in this study.

Strains	Relevant Genotype	Source
*Zymomonas mobilis* subsp. *mobilis* ATCC 29191	Wild type	ATCC 29191
*Lactobacillus johnsonii* ATCC 11506	Wild type	ATCC 11506
*Escherichia coli* NZY5α	*fhuA2* Δ(*argF^−^lacZ*)U169 *phoA glnV44*Φ80 Δ(*lacZ*)M15 *gyrA96 recA1 relA1 endA1 thi-1 hsdR17*	NZYTech (MB00401)
*E. coli* BL21 (DE3)	F^−^ *omp*T *gal dcm lon hsd*SB(rB- mB-) λ(DE3 *lacI lacUV5-T7 gene 1 ind1 sam7 nin5])	NZYTech (MB006)
*Saccharomyces cerevisiae* CEN.PK2-1C	*MATa ura3-52 his3*Δ*1 leu2-3*, *112 trp1-289 MAL2-8^c^ SUC2*	Euroscarf 30000A [26]
*S. cerevisiae* BY4741	*MATa his3*Δ*1 leu2*Δ*0 met15*Δ*0 ura3*Δ*0*	Euroscarf Y00000 [27]
Plasmids	Description	Source
pGEM^®^-T Easy	ColE1/pMB1/pBR322/pUC *ori*, *lacZ*, Amp^R^	Promega
pRSFDuet-1	RSF1030 *ori*, *lacI*, double P_T7*lac*_, Kan^R^	Novagen
pGEM_*Lbj*UGD	pGEM^®^-T Easy harboring *L. johnsonii* UGD (*Lbj*UGD) gene	This study
pRSFDuet_*Zm*UGD	pRSFDuet-1 carrying *Z. mobilis* UGD (*Zm*UGD) gene	This study
pRSFDuet_*Lbj*UGD	pRSFDuet-1 carrying *Lbj*UGD gene	This study
pUC57_*Ch*UGD	pMB1 *ori*, Amp^R^; pUC57 carrying *Capra hircus* UGD (*Ch*UGD) gene codon-optimized for *S. cerevisiae*	NZYTech
pSP-GM1	pUC *ori*, Amp^R^, 2 µ *ori*, *URA3* P*_TEF1_* P_PGK*1*_	Addgene #64739 [28]
p426GPD	pUC *ori*, Amp^R^, 2 µ *ori*, *URA3* P_GPD_	ATCC 87361
pSP-GM1_*Zm*UGD	pSP-GM1 carrying *Zm*UGD gene	This study
p426GPD_*Zm*UGD	p426GPD carrying *Zm*UGD gene	This study
pSP-GM1_*Lbj*UGD	pSP-GM1 carrying *Lbj*UGD gene	This study
p426GPD_*Lbj*UGD	p426GPD carrying *Lbj*UGD gene	This study
pSP-GM1_*Ch*UGD	pSP-GM1 carrying *Ch*UGD gene	This study
p426GPD_*Ch*UGD	p426GPD carrying *Ch*UGD gene	This study

**Table 2:** Kinetic parameters V_max_ and K_m_ for each enzyme expressed in this study. *Zm*UGD: Uridine diphosphate-glucose dehydrogenase (UGD) from *Zymomonas mobilis*; *Lbj*UGD: UGD from *Lactobacillus johnsonii*; *Ch*UGD: UGD from *Capra hircus*.

Expression System	Enzyme Expressed	V_max_ (μmol·min^−1^·g_protein_^−1^)	K_m_ (µM)	V_max_/K_m_ (L·min^−1^·g_protein_^−1^)
*Escherichia coli*	*Zm*UGD	81	2586	3.1 × 10^−2^
	*Lbj*UGD	66	1939	3.4 × 10^−2^
*Saccharomyces cerevisiae*	*Ch*UGD	2.2	841	2.6 × 10^−3^
	*Zm*UGD	3.7	1234	3.0 × 10^−3^
	*Lbj*UGD	1.4	314	4.5 × 10^−3^

## Data Availability

Not applicable.

## Supplementary Materials

The following are available online at https://www.mdpi.com/article/10.3390/life11111201/s1, Table S1: Primers used in this study, Table S2: Gene sequence of UGD from *Capra hircus*, Table S3: Results of BlastP sequences search against UniProtKB/Swiss-Prot database, Figure S1: Multiple amino acid sequence alignment of the three uridine diphosphate-glucose dehydrogenases used in this study, Figure S2: In vitro reduced nicotinamide adenine dinucleotide (NADH) production over time in engineered *Escherichia coli*, Figure S3: SDS-PAGE gels from *Saccharomyces cerevisiae* cell extracts, Figure S4: In vitro reduced nicotinamide adenine dinucleotide (NADH) production over time in engineered *S. cerevisiae.*
