# Supplementary material for: Cloning, Expression and Characterization of UDP-Glucose Dehydrogenases
Source: Life (Basel). 2021 Nov 7;11(11):1201. doi: 10.3390/life11111201 (PMC8617651; doi:10.3390/life11111201)
Supplement: Supplementary file 1 [file life-11-01201-s001.zip › life-1438817-supplementary.pdf]

Cloning, Expression and Characterization of UDP-glucose Dehydrogenases

Márcia R Couto, Joana L Rodrigues* and Lígia R Rodrigues

Centre of Biological Engineering, University of Minho, Braga 4710-057, Portugal

*Corresponding author: joanarodrigues@ceb.uminho.pt

Supplementary Information

Table S1. Primers used in this study for gene amplification, colony PCR and sequencing. Histidine Tag sequence is underlined.

**Primers**	**Template**	**Sequence (5' → 3')**	**Restriction enzyme**
Gene amplification for cloning			
Zm_Fw	*Zymomonas mobilis* gDNA	AAAGGATCCAAAAAAAATGCATCACCATCACCATCACCGCATTACCATGATCGGTTCC	*Bam*HI
Zm_Rv		AAAACTCGAGTTACCCTGCATTGGGTCGAC	*Xho*I
Lbj_Fw	*Lactobacillus. johnsonii* gDNA	AAAGGATCCAAAAAAAATGCATCACCATCACCATCACAATTCATATAAAATTGCAGTTG	*Bam*HI
Lbj_Rv		AAAACTCGAGCTAATCTTTTCTAAAAATATCTCTTG	*Xho*I
Zm_prsf_Fw	p426GPD_*Zm*UGD	AAAGAATTCTATGCGCATTACCATGATCGGTTC	*Eco*RI
Zm_prsf_Rv		AAAGCGGCCGCTTACCCTGCATTGGGTCGAC	*Not*I
Lbj_prsf_Fw	pGEM_*Lbj*UGD	AAAAAGAATTCTATGAATTCATATAAAATTGCAGTTG	*Eco*RI
Lbj_prsf_Rv		AAAGCGGCCGCCTAATCTTTTCTAAAAATATCTCTTG	*Not*I
Ch_Fw	pUC19_*Ch*UGD	AAAGGATCCAAAAAAAATGCATCACCATCACCATCACTTTGAGATTAAAAAAATCTGC	*Bam*HI
Ch_Rv		AAAACTCGAGCTATACTCTCGGTTTCTTATTC	*Xho*I
Colony PCR and Sequencing			
M13_Fw	pGEM®-T Easy and derivatives	GTAAAACGACGGCCAGT	
M13_Rv		GCGGATAACAATTTCACACAGG	
pRSFDuet_Fw	pRSFDuet-1 and derivatives	GGATCTCGACGCTCTCCCTT	
T7 terminator _Rv		CTAGTTATTGCTCAGCGGT	
PGK1p_Fw	pSP-GM1 and derivatives	GTGATCTCCAGAGCAAAGT	
CYCt_Rv		CTTCGAGCGTCCCAAAAC	
p426seq_Fw	p426GPD and derivatives	ACTAAAGGGAACAAAAGCTG	
CYC 1_Rv		ACTTCAGGTTGTCTAACTCC	

Table S2. Gene sequence of UGD from *Capra hircus* codon optimized (NZYTech) for *Saccharomyces cerevisiae*

**>ugd1 (UDP-glucose 6-dehydrogenase) from *C. hircus* (1485 bp; GC%: 41.89; CAI: 0.74)**
ATGTTTGAGATTAAAAAAATCTGCTGCATCGGTGCGGGTTATGTCGGTGGTCCTACTTGT<br>AGCGTTATTGCACACATGTGTCCTGAAATTCGTGTCACCGTCGTTGACATCAATGAGTCA<br>AGAATAAATGCTTGGAATAGTCCGACTCTACCAATTTATGAACCCGGTTTGAAAGAAGT<br>AGTTGAATCTTGCAGAGGTAAGAACTTGTTTTTCTCTACAAACATCGACGACGCGATAAA<br>GGAAGCTGATCTGGTATTCATCTCTGTTAATACACCCACCAAAACGTACGGTATGGGCAA<br>GGGTAGAGCTGCTGATCTAAAGTACATCGAAGCGTGTGCACGTCGAATAGTTCAGAACT<br>CTCATGGTTACAAGATCGTGACAGAAAAGTCCACAGTTCCAGTTAGAGCAGCTGAATCC<br>ATAAGGAGGATCTTCGATGCTAACACAAAGCCAAACTTGAACTTACAGGTATTGTCAAA<br>CCCGGAATTTTTAGCTGAAGGGACGGCCATCAAGGATTTGAAGAACCCAGACCGAGTTC<br>TAATCGGTGGTGACGAGACACCTGAAGGTCAAAGAGCTGTCCAAGCTCTCTGTGCAGTTT<br>ACGAGCATTGGGTGCCCCGTGAAAAAATTCTTACAACCAACACTTGGTCTTCAGAGTTGT<br>CGAAACTAACCGCTAATGCATTTTTAGCTCAAAGAATTTCTAGCATCAATTCAATTAGTG<br>CCTTGTGTGAAGCAACCGGCGCAGATGTAGAGGAAGTCGCTACAGCTATTGGAATGGAT<br>CAAAGAATAGGTTCTAAATTTCTAAAAGCTAGCGTAGGATTTGGTGGTTCTTGCTTTCAA<br>AAGGATGTCCTTAATTTAGTGTATTTGTGTGAAGCCCTTAATTTACCAGAAGTCGCTAGAT<br>ACTGGCAACAGGTCATAGATATGAACGACTATCAACGTAGAAGATTCGCTTCGAGGATA<br>ATTGATTCTCTATTTAACACAGTTACTGATAAAAAGATTGCCATATTGGGATTCGCCTTTA<br>AGAAAGACACTGGCGACACAAGAGAGTCATCATCAATATACATTTCTAAGTATTTGATG<br>GATGAAGGTGCTCACTTGCATATTTATGATCCAAAGGTGCCACGTGAACAGATTGTGGTA<br>GATTTATCGCATCCCGGCGTTAGCAAGGATGATCAAGTTGCTCGTCTTGTGACAATCTCC<br>AAAGACCCATACGAAGCATGTGATGGTGCTCACGCAGTGGTTATTTGTACAGAATGGGA<br>CATGTTTAAAGAACTGGATTACGAGAGAATACACAAAAAAATGTTGAAGCCCGCTTTTA<br>TTTTTGATGGTAGGAGAGTGTTGGACGGGCTACATAACGAATTGCAAACCATAGGCTTTC<br>AAATTGAGACAATTGGGAAGAAGGTTTCCAGTAAAAGAATCCCTTATGCGCCGTCAGGG<br>GAGATACCAAAGTTTAGTCTACAAGACATGCCGAATAAGAAACCGAGAGTATAG

Table S3. Results of blastp sequences search against UniProtKB/Swiss-Prot database ordered by the identity percentages obtained. Results with score <50 were removed to exclude enzymes that showed similarity with only one of the protein domains

Query	Organism	Protein	Access Number	Identity (%)
*Zm*UGD	*Sinorhizobium meliloti* 1021	UGD	O54068.2	67.05
	Bacillus subtilis subsp. *subtilis* str. 168	UGD (YwqF)	P96718.1	48.51
	Bacillus subtilis subsp. *subtilis* str. 168	UGD (TuaD)	O32271.1	46.67
	Pseudomonas aeruginosa PAO1	UGD	O86422.2	45.86
	Rickettsia bellii RML369-C	UGD	Q1RKF8.1	44.93
	Methanocaldococcus jannaschii DSM 2661	UGD	Q58454.1	44.04
	Rickettsia conorii str. Malish 7	UGD	Q92GB1.1	44.01
	Rickettsia felis URRWXCal2	UGD	Q4UK39.1	43.91
	Rickettsia typhi str. Wilmington	UGD	Q68VX0.1	43.78
	Rickettsia prowazekii str. Madrid E	UGD	O05973.1	43.09
	Bacillus subtilis subsp. *subtilis* str. 168	Putative UGD (YtcA)	O34862.1	40.50
	Haloferax volcanii DS2	UGD (AglM)	D4GYH5.1	39.00
	Drosophila melanogaster	UGD	O02373.1	37.53
	Pseudomonas aeruginosa PAO1	GMD	P11759.2	36.91
	Homo sapiens	UGD	O60701.1	36.85
	Pongo abelii	UGD	Q5R7B3.1	36.85
	Bos taurus	UGD	P12378.2	36.64
	Mus musculus	UGD	O70475.1	36.64
	Oryza sativa Japonica Group	UGD1	Q75GS4.1	36.46
	Glycine max	UGD1	Q96558.1	36.23
	Gallus gallus	UGD	Q5F3T9.1	36.23
	Oryza sativa Japonica Group	UGD2	B7F958.1	36.09
	Rattus norvegicus	UGD	O70199.1	36.01
	Arabidopsis thaliana	UGD2	Q9LIA8.1	35.96

	Arabidopsis thaliana	UGD3	Q9LF33.1	35.73
	Arabidopsis thaliana	UGD1	Q9FZE1.1	35.39
	Caenorhabditis elegans	UGD	Q19905.1	35.36
	Arabidopsis thaliana	UGD4	Q9FM01.1	35.31
	Azotobacter vinelandii	GMD	P51585.1	35.08

Table S3. Results of blastp sequences search against UniProtKB/Swiss-Prot database ordered by the identity percentages obtained. Results with score <50 were removed to exclude enzymes that showed similarity with only one of the protein domains (*continuation*)

**Query**	**Organism**	**Protein**	**Access Number**	**Identity (%)**
*Zm*UGD (cont.)	*Oryza sativa* Japonica Group	UGD5	Q2QS13.1	35.02
	Pseudomonas syringae pv. tomato str. DC3000	GMD	Q887P8.1	34.99
	Oryza sativa Japonica Group	UGD3	Q9AUV6.1	34.75
	Oryza sativa Japonica Group	UGD4	Q2QS14.1	34.53
	Pseudomonas putida KT2440	GMD	Q88NC4.1	33.18
	Pseudomonas syringae pv. syringae	GMD	P59793.1	32.60
	Escherichia coli K5	UGD	Q47329.1	32.39
	Pseudomonas savastanoi pv. phaseolicola	GMD	O07299.1	31.56
	Escherichia coli K-12	UAMAD	P27829.4	31.01
	Escherichia coli O8:K40	UGD	O33952.1	30.79
	Salmonella enterica subsp. *enterica serovar Typhi*	UAMAD	Q8Z389.1	30.66
	Salmonella enterica subsp. *enterica serovar Typhimurium* str. LT2	UAMAD	Q9L6R4.1	30.39
	Streptococcus pneumoniae	UGD	Q57346.2	29.92
	Salmonella enterica subsp. *enterica serovar Typhimurium* str. LT2	UGD	Q04873.1	29.86
	Shigella flexneri	Putative UGD	P37791.1	29.56
	Escherichia coli O157:H7	UGD	Q7DBF9.1	29.21
	Escherichia coli CFT073	UGD	Q8FG45.1	29.21
	Escherichia coli K-12	UGD	P76373.1	29.21

	Escherichia coli O111:H-	UGD	Q04872.1	29.13
	Methanococcus maripaludis S2	UAMAD	Q6LZC3.1	28.70
	Streptococcus pyogenes	UGD	P0C0F4.1	28.57
	Streptococcus pyogenes serotype M1	UGD	P0C0F5.1	28.57
	Methanococcus maripaludis C7	UAMAD	A6VK13.1	28.47
	Streptococcus pyogenes SSI-1	UGD	P0DG69.1	28.30
	Streptococcus pyogenes MGAS315	UGD	P0DG68.1	28.30
	Methanococcus maripaludis C5	UAMAD	A4FY94.1	28.02
	Ralstonia solanacearum GMI1000	NAGAAD	P58591.1	27.87

Table S3. Results of blastp sequences search against UniProtKB/Swiss-Prot database ordered by the identity percentages obtained. Results with score <50 were removed to exclude enzymes that showed similarity with only one of the protein domains (*continuation*)

Query	Organism	Protein	Access Number	Identity (%)
*Zm*UGD (cont.)	*Yersinia pestis*	UAMAD	Q8ZAE4.1	27.85
	Methanocaldococcus jannaschii DSM 2661	UAMAD	Q57871.1	27.52
	Ralstonia solanacearum	NAGAAD	Q45410.1	27.17
	Methanococcus vannielii SB	UAMAD	A6USK4.1	26.90
	Methanococcus aeolicus Nankai-3	UAMAD	A6UU98.1	26.09
	Pseudomonas aeruginosa PAO1	UAGAD	G3XD94.1	25.74
*Lbj*UGD	*Escherichia coli* O111:H-	UGD	Q04872.1	61.80
	Escherichia coli O8:K40	UGD	O33952.1	61.56
	Escherichia coli CFT073	UGD	Q8FG45.1	60.34
	Escherichia coli O157:H7	UGD	Q7DBF9.1	60.10
	Escherichia coli K-12	UGD	P76373.1	59.85
	Salmonella enterica subsp. *enterica serovar Typhimurium* str. LT2	UGD	Q04873.1	59.61
	Streptococcus pyogenes serotype M1	UGD	P0C0F5.1	57.77
	Streptococcus pyogenes WF50	UGD	P0C0F4.1	57.77
	Streptococcus pneumoniae	UGD	Q57346.2	57.66
	Escherichia coli K5	UGD	Q47329.1	57.63

Streptococcus pyogenes MGAS315	UGD	P0DG68.1	57.52
Streptococcus pyogenes SSI-1	UGD	P0DG69.1	57.52
Shigella flexneri	Putative UGD	P37791.1	57.42
Methanocaldococcus jannaschii DSM 2661	UGD	Q58454.1	32.85
Rickettsia prowazekii str. Madrid E	UGD	O05973.1	30.42
Bacillus subtilis subsp. *subtilis* str. 168	UGD (YwqF)	P96718.1	30.03
Rickettsia bellii RML369-C	UGD	Q1RKF8.1	30.02
Rickettsia typhi str. Wilmington	UGD	Q68VX0.1	29.71
Bacillus subtilis subsp. *subtilis* str. 168	UGD (TuaD)	O32271.1	29.64
Rickettsia felis URRWXCal2	UGD	Q4UK39.1	29.10
Sinorhizobium meliloti 1021	UGD	O54068.2	29.06
Bacillus subtilis subsp. *subtilis* str. 168	Putative UGD (YtcA)	O34862.1	28.76
Rickettsia conorii str. Malish 7	UGD	Q92GB1.1	28.46

Table S3. Results of blastp sequences search against UniProtKB/Swiss-Prot database ordered by the identity percentages obtained. Results with score <50 were removed to exclude enzymes that showed similarity with only one of the protein domains (*continuation*)

Query	Organism	Protein	Access Number	Identity (%)
*Lbj*UGD (cont.)	*Azotobacter vinelandii*	GMD	P51585.1	28.39
	Pseudomonas syringae pv. Syringae	GMD	P59793.1	28.00
	Pseudomonas syringae pv. tomato str. DC3000	GMD	Q887P8.1	27.93
	Pseudomonas savastanoi pv. phaseolicola	GMD	O07299.1	27.76
	Arabidopsis thaliana	UGD4	Q9FM01.1	27.11
	Caenorhabditis elegans	UGD	Q19905.1	27.03
	Pseudomonas aeruginosa PAO1	GMD	P11759.2	26.77
	Bacillus licheniformis DSM 13 = ATCC 1458	GPD	Q65I16.1	26.53
	Rattus norvegicus	UGD	O70199.1	26.51
	Homo sapiens	UGD	O60701.1	26.51
	Bos taurus	UGD	P12378.2	26.51

	Pseudomonas aeruginosa PAO1	UGD	O86422.2	26.37
	Haloferax volcanii DS2	UGD (AglM)	D4GYH5.1	26.32
	Pongo abelii	UGD	Q5R7B3.1	26.25
	Mus musculus	UGD	O70475.1	26.25
	Pseudomonas putida KT2440	GMD	Q88NC4.1	25.85
	Drosophila melanogaster	UGD	O02373.1	25.39
	Staphylococcus aureus	UGD/GMD superfamily (CapL)	P39861.1	25.24
*Ch*UGD	*Bos taurus*	UGD	P12378.2	99.80
	Homo sapiens	UGD	O60701.1	98.38
	Pongo abelii	UGD	Q5R7B3.1	98.18
	Mus musculus	UGD	O70475.1	96.36
	Rattus norvegicus	UGD	O70199.1	96.15
	Gallus gallus	UGD	Q5F3T9.1	92.51
	Drosophila melanogaster	UGD	O02373.1	68.55
	Caenorhabditis elegans	UGD	Q19905.1	65.87
	Glycine max	UGD1	Q96558.1	61.75
	Arabidopsis thaliana	UGD4	Q9FM01.1	61.75

Table S3. Results of blastp sequences search against UniProtKB/Swiss-Prot database ordered by the identity percentages obtained. Results with score <50 were removed to exclude enzymes that showed similarity with only one of the protein domains (*continuation*)

Query	Organism	Protein	Access Number	Identity (%)
*Ch*UGD (cont.)	*Oryza sativa* Japonica Group	UGD5	Q2QS13.1	61.44
	Arabidopsis thaliana	UGD2	Q9LIA8.1	61.32
	Arabidopsis thaliana	UGD1	Q9FZE1.1	61.31
	Arabidopsis thaliana	UGD3	Q9LF33.1	61.11
	Oryza sativa Japonica Group	UGD4	Q2QS14.1	61.02
	Oryza sativa Japonica Group	UGD2	B7F958.1	60.98
	Oryza sativa Japonica Group	UGD3	Q9AUV6.1	60.81

	Oryza sativa Japonica Group	UGD1	Q75GS4.1	59.75
	Sinorhizobium meliloti 1021	UGD	O54068.2	37.96
	Bacillus subtilis subsp. *subtilis* str. 168	UGD (YwqF)	P96718.1	35.42
	Methanocaldococcus jannaschii DSM 2661	UGD	Q58454.1	35.38
	Bacillus subtilis subsp. *subtilis* str. 168	UGD (TuaD)	O32271.1	34.77
	Haloferax volcanii DS2	UGD (AglM)	D4GYH5.1	34.68
	Pseudomonas aeruginosa PAO1	UGD	O86422.2	32.70
	Rickettsia conorii str. Malish 7	UGD	Q92GB1.1	32.54
	Rickettsia felis URRWXCal2	UGD	Q4UK39.1	32.47
	Rickettsia typhi str. Wilmington	UGD	Q68VX0.1	32.47
	Rickettsia prowazekii str. Madrid E	UGD	O05973.1	32.40
	Rickettsia bellii RML369-C	UGD	Q1RKF8.1	32.26
	Bacillus subtilis subsp. *subtilis* str. 168	Putative UGD (YtcA)	O34862.1	31.29
	Pseudomonas syringae pv. syringae	GMD	P59793.1	29.17
	Pseudomonas syringae pv. tomato str. DC3000	GMD	Q887P8.1	29.09
	Pseudomonas savastanoi pv. phaseolicola	GMD	O07299.1	28.91
	Azotobacter vinelandii	GMD	P51585.1	28.68
	Pseudomonas aeruginosa PAO1	GMD	P11759.2	27.98
	Shigella flexneri	Putative UGD	P37791.1	27.82
	Salmonella enterica subsp. *enterica serovar Typhimurium* str. LT2	UGD	Q04873.1	27.72

Table S3. Results of blastp sequences search against UniProtKB/Swiss-Prot database ordered by the identity percentages obtained. Results with score <50 were removed to exclude enzymes that showed similarity with only one of the protein domains (*continuation*)

**Query**	**Organism**	**Protein**	**Access Number**	**Identity (%)**
*Ch*UGD (cont.)	*Methanococcus maripaludis* C7	UAMAD	A6VK13.1	27.47
	Escherichia coli O8:K40	UGD	O33952.1	27.37

	Methanococcus maripaludis S2	UAMAD	Q6LZC3.1	27.25
	Escherichia coli O111:H-	UGD	Q04872.1	27.25
	Methanocaldococcus jannaschii DSM 2661	UAMAD	Q57871.1	27.21
	Pseudomonas putida KT2440	GMD	Q88NC4.1	27.08
	Methanococcus aeolicus Nankai-3	UAMAD	A6UU98.1	26.87
	Methanococcus vannielii SB	UAMAD	A6USK4.1	26.82
	Escherichia coli K5	UGD	Q47329.1	26.53
	Escherichia coli O157:H7	UGD	Q7DBF9.1	26.36
	Methanococcus maripaludis C5	UAMAD	A4FY94.1	26.18
	Escherichia coli CFT073	UGD	Q8FG45.1	26.09
	Escherichia coli K-12	UGD	P76373.1	26.09
	Pyrococcus horikoshii OT3	UAMAD	O59284.1	25.74
	Streptococcus pneumoniae	UGD	Q57346.2	25.68
	Salmonella enterica subsp. *enterica serovar Typhi*	UGD/GMD superfamily (VipA/TviB)	Q04972.1	23.79

Enzyme abbreviations: UGD, uridine diphosphate(UDP)-glucose 6-dehydrogenase; GMD, guanosine diphosphate(GDP)-mannose 6-dehydrogenase; UAMAD, UDP-*N*-acetyl-D-mannosamine dehydrogenase; UAGAD, UDP-*N*-acetylglucosamine 6-dehydrogenase; and NAGAAD, nucleoside diphosphate(NDP)-*N*-acetyl-D-galactosaminuronic acid dehydrogenase.

```
               NAD-binding domain
Lajo   -MNSYKIAVAGTGYVG---LSLATLLSQHNEVTAVDIVPEKVDLINNHKSPIVDKEIEDF   56
Cahi   MFEIKKICCIGAGYVGGPTCSVIAHMCPEIRVTVVDINESRINAWNSPTLPIYEPGLKEV   60
Zymo   ----MRITMIGSGYVG--LVSGACFSDFGHNVICVDHDQEKIALLQKGVMPIYEPGLADM   54
            :*   *:****    *         .*  **   .::   :.   ** :  : :.

               NAD-binding domain
Lajo   LANKD--LNLKATTDAESAYKDADFVIIATPTNYDSE----KNFFDTSAVEAVIKLVKQY   110
Cahi   VESCR-GKNLFFSTNIDDAIKEADLVFISVNTPTKTYGMGKGRAADLKYIEACARRIVQN   119
Zymo   VANNVKAGRLSFSNDLASSVKNADAVFIAVGTPSRRG----DGHADLSYVFAAAKEIAAS   110
       : .     .*  :.:  .: *:** *:*:. *             * . : *  : :

               NAD-binding domain
Lajo   NPQA-IMIIKSTIPVGYTNHVRKEFE-------TENIIFSPEFLREGHALYDNLHPSRIV   162
Cahi   SHGYKIVTEKSTVPVRAAESIRRIFDANTKPNLNLQVLSNPEFLAEGTAIKDLKNPDRVL   179
Zymo   VSDNTVVVDKSTVPVGTGDEVERIIREIAPDK-KIWVASNPEFLREGAAIGDFKRPDRIV   169
            ::  ***:**   : :.: :        .  : .**** ** *: *  .*.*::

       NAD-binding domain                      Central domain
Lajo   VGTDK---ENTKLVEAAHTFASLLQEGAEDKDIATLFMGFTEAEAVKLFANTYLALRVSY   219
Cahi   IGGDETPEGQRAVQALCAVYEHWVP------REKILTTNTWSSELSKLTANAFLAQRISS   233
Zymo   IGTENP----EAREIMQEIYRPLYLN-----QSPILFTSRRSAELIKYASNAFLATKITF   220
       :* ::              :               *  .  .:*  *  :*::** :::

               Central domain
Lajo   FNELDTYAESKGLNTQEIIDGVGLDPRIGSHYNNPSFGYGGYCLPKDTKQLLANYKDV--   277
Cahi   INSISALCEATGADVEEVATAIGMDQRIGSKFLKASVGFGGSCFQKDVLNLVYLCEALNL   293
Zymo   INEMADLCEAVGGDVQDIARGIGADNRIGSKFLHAGPGYGGSCFPKDTLALLKTAENF--   278
       :*.:   .*: * :.:::  .:* * ****:: : . *:** *: **.  *:   : .

       Central domain
Lajo   --PENLIEAIVKSNDTRKDFIADQVLSKAGYYDYDDNNTYDPSEEKQVTIGVYRLTMKSN   335
Cahi   PEVARYWQQVIDMNDYQRRRFASRIIDSLFNT------------VTDKKIAILGFAFKKD   341
Zymo   ATPLRVVESVVTANNIRKRAMARKVLQAIGKD------------MRGQKIGLLGLTFKPN   326
           .  : ::  *: ::  :* :::.                     .*.:  :::* :

               UDP-binding domain
Lajo   SDNFRQSSVQGVMKRVKAKGAKIIIFEPTLENGSTF------------------------   371
Cahi   TGDTRESSSIYISKYLMDEGAHLHIYDPKVPREQIVVDLSHPGVSKDDQVARLVTISKDP   401
Zymo   TDDMRDAPSLALIQGLEDAGAIIHAYDPEGMTQ---ARPLLPNVIF----------EDEP   373
       :.: *::    : : :   ** :  ::*

               UDP-binding domain
Lajo   -------FGSEVVNDLDEFKNKSDAIIAN---------RYNTILDDVKDKVYTRDIFRKD   415
Cahi   YEACDGAHAVVICTEWDMFKELDYERIHKKMLKPAFIFDGRRVLDGLHNELQTIGFQIET   461
Zymo   YKVAEKAEALVIVTEWDAFRALDLKRIAGLLKTP-LLLDLRNIYTE--EEAKKAGLVYHG   430
               .  : .: * *:  .   *             . :     ::  . .:  .

Lajo   ------------------------------------   415
Cahi   IGKKVSSKRIPYAPSGEIPKFSLQDMPNKKPRV   494
Zymo   IGRPNAG--------------------------   437
```

Figure S1. Multiple amino acid sequence alignment of the three uridine diphosphate (UDP)-glucose dehydrogenases used in this study generated by Clustal Omega (1.2.4) (EMBL-EBI). Species are abbreviated as the first two letters of the genus followed by the two first letters of the species: Lajo, *Lactobacillus johnsonii*; Cahi, *Capra hircus*; and Zymo, *Zymomonas mobilis*. An asterisk (*) marks the residues that are conserved in the three sequences; a colon (:) indicates conservation between groups of strongly similar properties (scoring > 0.5 in the Gonnet Point Accepted Mutation (PAM) 250

matrix); and a period (.) indicates conservation between groups of weakly similar properties (scoring =< 0.5 in the Gonnet PAM 250 matrix). Conserved amino acids within 63 members of UDP-glucose 6-dehydrogenase/ guanosine diphosphate (GDP)-mannose 6-dehydrogenase (UGD/GMD) superfamily are highlighted in grey while conserved sites within only UGD enzymes are highlighted in yellow. The nicotinamide adenine dinucleotide (NAD)-binding domain contains a characteristic dinucleotide binding Rossman fold, indicated in a blue box, composed by a conserved glycine-rich phosphate-binding loop with the pattern GXGXXG linking the first β-sheet to the following α-helix that, in the case of UGD/GMD dehydrogenase enzymatic family, has a strictly conserved tyrosine so that the region becomes GXGYXG. The central domain is a long α-helical region that serves as the dimerization interface for these enzymes. The C-terminal substrate-binding domain forms an incomplete dinucleotide binding fold, primarily responsible for binding the UDP moiety of the UDP-sugar in a deep pocket. The described active site of UGD contains residues from the three domains that are highly conserved within the superfamily and include: N-terminal threonine (T_{122}), central α-helix lysine, (K_{207}), and asparagine, (N_{211}) and C-terminal contributes with cysteine (C_{263}) and aspartic acid (D_{267}) (reference sites from *Z. mobilis* sequence)

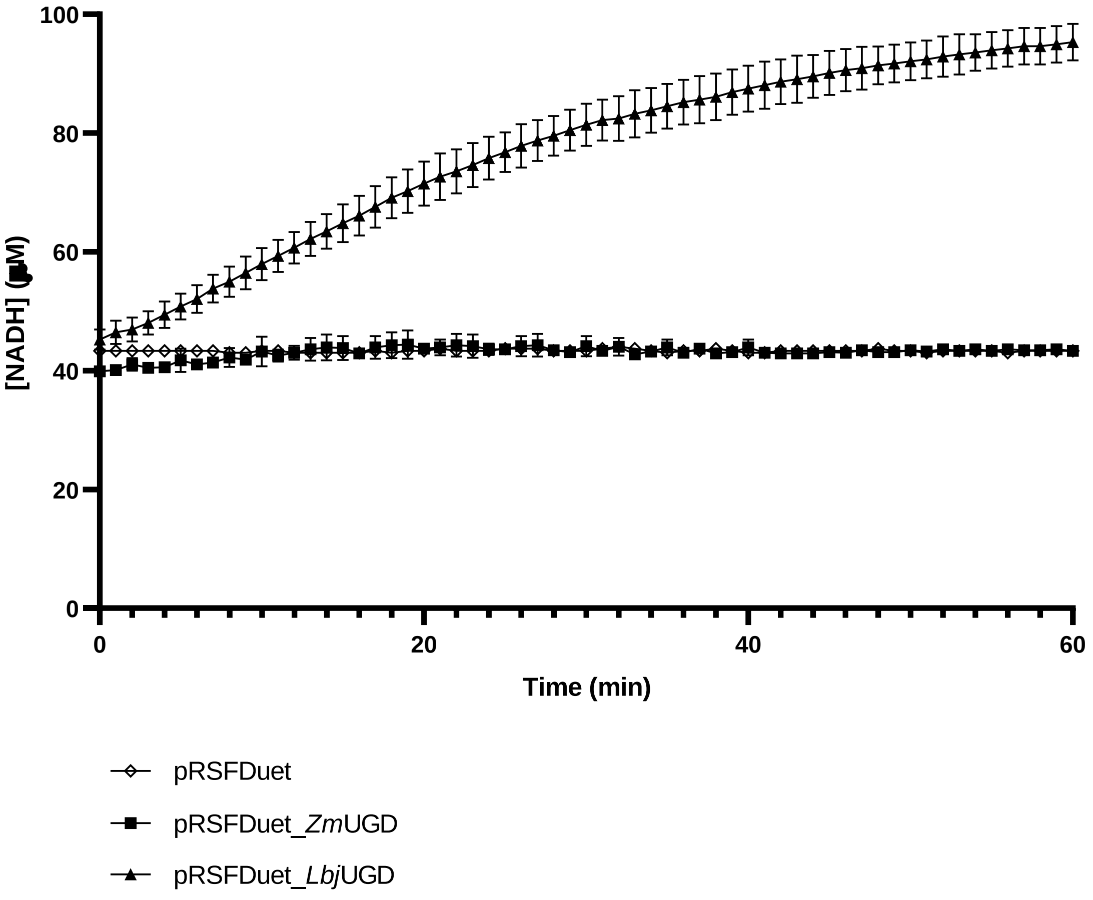

Figure S2. *In vitro* reduced nicotinamide adenine dinucleotide (NADH) production over time in engineered *Escherichia coli* at pH 7.2 as obtained by monitoring absorbance at 340 nm.

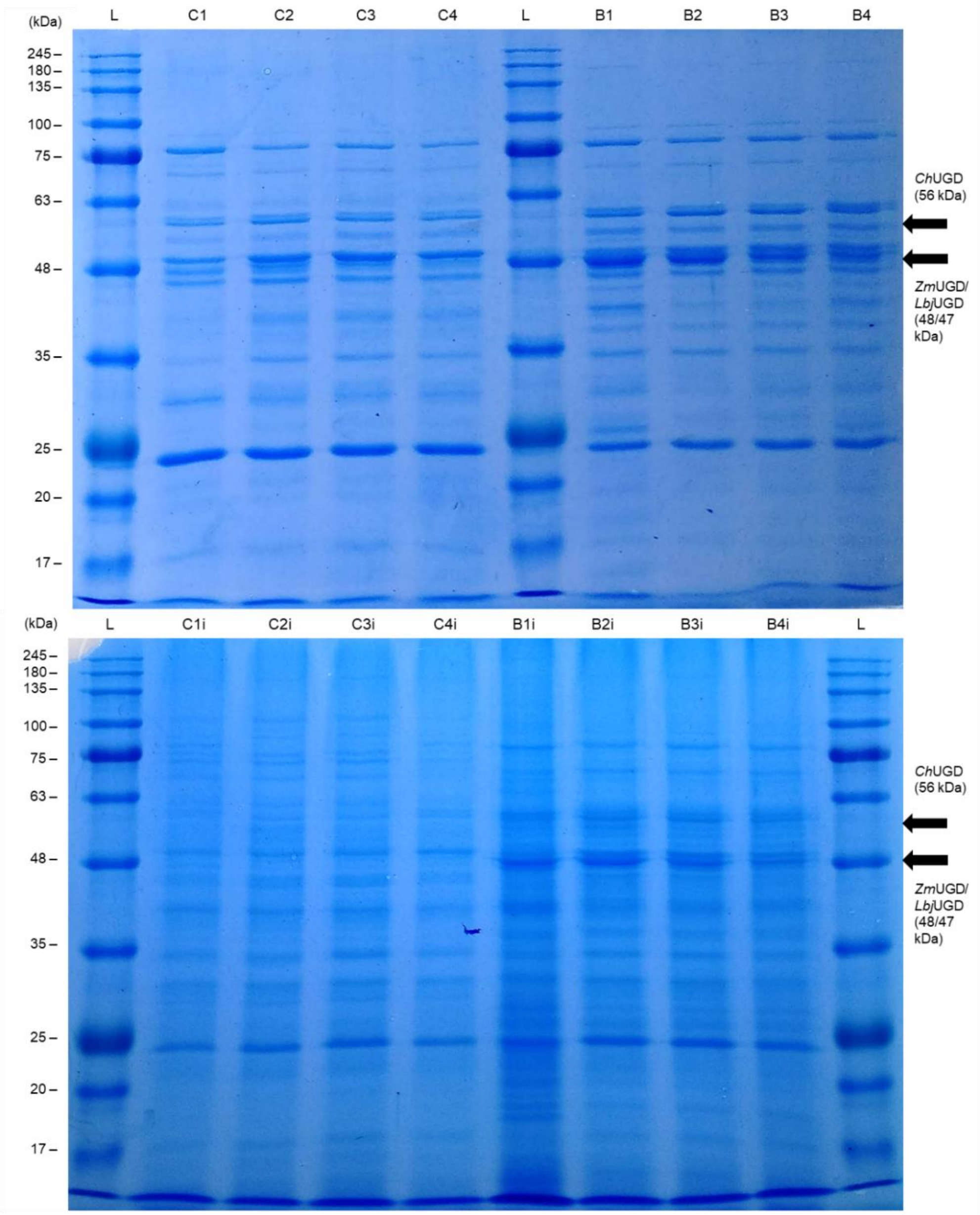

Figure S3. Sodium dodecyl sulfate polyacrylamide gel electrophoresis (SDS-PAGE) gels from *Saccharomyces cerevisiae* cell extracts (15 µg). In the upper gel, the samples loaded were: cell extract from *S. cerevisiae* CEN.PK2-1C wild-type (C1), cell extract from *S. cerevisiae* CEN.PK2-1C expressing *Ch*UGD (C2) cell extract from *S. cerevisiae* CEN.PK2-1C expressing *Zm*UGD (C3), cell extract from *S. cerevisiae* CEN.PK2-1C expressing *Lbj*UGD (C4), *S. cerevisiae* BY4741 wild-type (B1), *S. cerevisiae* BY4741 expressing *Ch*UGD (B2), *S. cerevisiae* BY4741 expressing *Zm*UGD (B3) and *S. cerevisiae* BY4741 expressing *Lbj*UGD (B4). The lower gel shows the proteins in the corresponding insoluble phases. L: NZYColour Protein Marker II ladder.

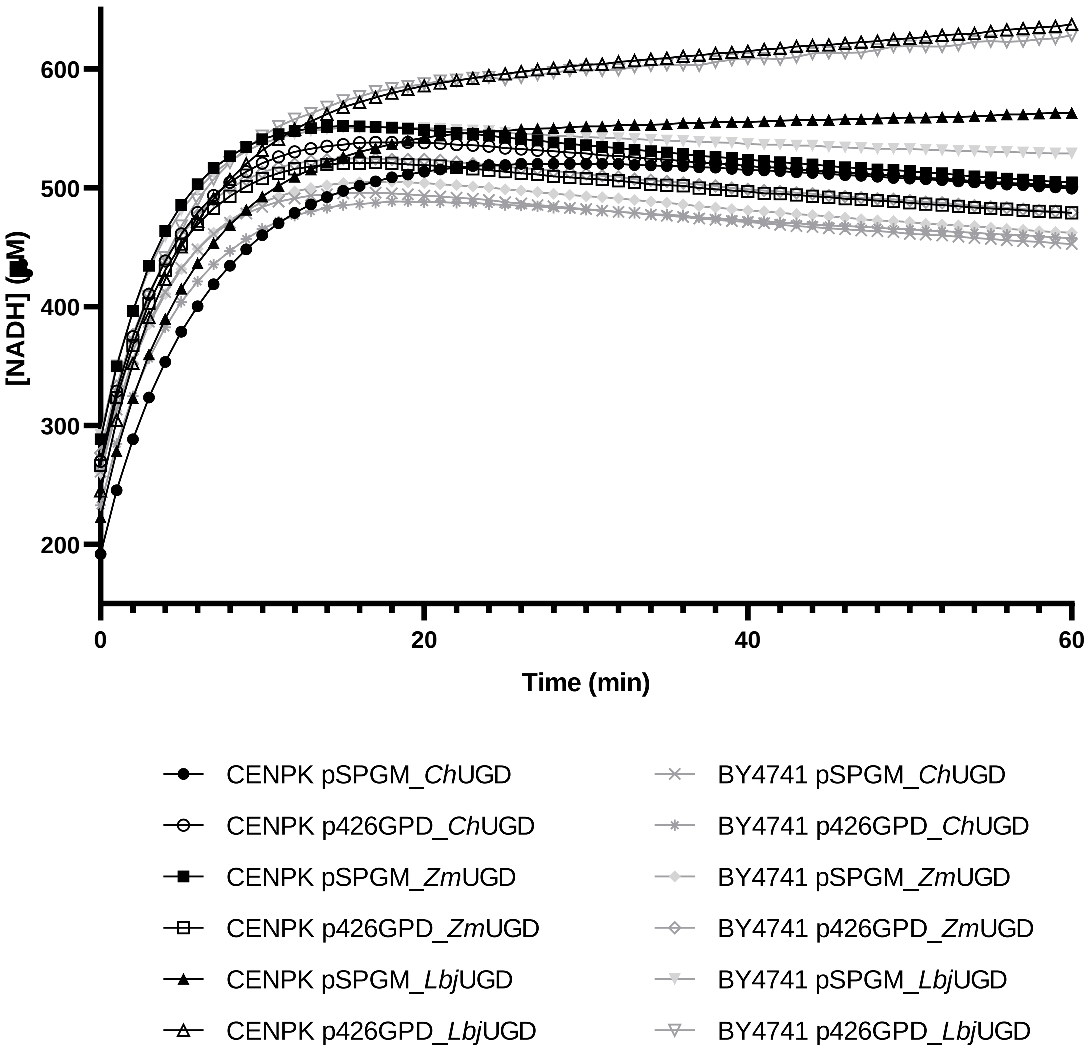

Figure S4. *In vitro* reduced nicotinamide adenine dinucleotide (NADH) production over time in engineered *S. cerevisiae* yeasts at pH 9.2 obtained by monitoring absorbance at 340 nm.
